# Prion Infections and Anti-PrP Antibodies Trigger Converging Neurotoxic Pathways

**DOI:** 10.1371/journal.ppat.1004662

**Published:** 2015-02-24

**Authors:** Uli S. Herrmann, Tiziana Sonati, Jeppe Falsig, Regina R. Reimann, Paolo Dametto, Tracy O’Connor, Bei Li, Agnes Lau, Simone Hornemann, Silvia Sorce, Uli Wagner, Despina Sanoudou, Adriano Aguzzi

**Affiliations:** 1 Institute of Neuropathology, University Hospital of Zurich, Zurich, Switzerland; 2 Institute of Surgical Pathology, University Hospital of Zurich, Zurich, Switzerland; 3 Department of Pharmacology, Medical School, University of Athens, Athens, Greece; University of Edinburgh, UNITED KINGDOM

## Abstract

Prions induce lethal neurodegeneration and consist of PrP^Sc,^ an aggregated conformer of the cellular prion protein PrP^C.^ Antibody-derived ligands to the globular domain of PrP^C^ (collectively termed GDL) are also neurotoxic. Here we show that GDL and prion infections activate the same pathways. Firstly, both GDL and prion infection of cerebellar organotypic cultured slices (COCS) induced the production of reactive oxygen species (ROS). Accordingly, ROS scavenging, which counteracts GDL toxicity *in vitro* and *in vivo*, prolonged the lifespan of prion-infected mice and protected prion-infected COCS from neurodegeneration. Instead, neither glutamate receptor antagonists nor inhibitors of endoplasmic reticulum calcium channels abolished neurotoxicity in either model. Secondly, antibodies against the flexible tail (FT) of PrP^C^ reduced neurotoxicity in both GDL-exposed and prion-infected COCS, suggesting that the FT executes toxicity in both paradigms. Thirdly, the PERK pathway of the unfolded protein response was activated in both models. Finally, 80% of transcriptionally downregulated genes overlapped between prion-infected and GDL-treated COCS. We conclude that GDL mimic the interaction of PrP^Sc^ with PrP^C,^ thereby triggering the downstream events characteristic of prion infection.

## Author Summary

Prion diseases are a group of infectious, invariably fatal neurodegenerative diseases. Progress in developing therapeutics is slow, partly because animal models of prion diseases require stringent biosafety and are very slow. We recently found that treatment of cerebellar slices with antibodies targeting the globular domain (GD ligands) of the prion protein (PrP) is neurotoxic. Here we compared this model to prion infection, and describe striking similarities. Both models involved the production of reactive oxygen species, and antioxidants could reverse the toxicity in cerebellar slices and even prolong the survival time of prion-infected mice. Antibodies targeting the flexible tail of PrP that prevent toxicity of GD ligands reduced the toxicity induced by prions. Endoplasmic reticulum stress, which is involved in prion toxicity, is also found in GD-ligand induced neurotoxicity. Finally, changes of gene expression were similar in both models. We conclude that prion infection and GD ligands use converging neurotoxic pathways. Because GD ligands induce toxicity within days rather than months and do not pose biosafety hazards, they may represent a powerful tool for furthering our understanding of prion pathogenesis and also for the discovery of antiprion drugs.

## Introduction

Prion diseases are lethal infectious diseases that propagate through the conversion of the cellular prion protein (PrP^C^) into a pathological conformer, the scrapie-associated prion protein (PrP^Sc^) [1]. Neuronal expression of PrP^C^ is required to mediate the neurotoxicity of PrP^Sc^ [2] and possibly also of other protein aggregates [3], yet the pathways leading to neurotoxicity are largely unknown. While caspase activation, autophagy, and Ca^2+^ dysregulation have been shown to occur after prion infections [4,5], ablation of Bax and caspase-12, or overexpression of Bcl-2, does not delay incubation time of prion-infected animals [6,7]. Induction of autophagy, despite enhancing PrP^Sc^ clearance *in vitro* and *in vivo*, did not prolong survival time of prion-infected mice [8]. Furthermore, excessive unfolded protein responses (UPR) in the endoplasmic reticulum (ER) plays a significant role in the pathogenesis of prion and other neurodegenerative diseases [9,10], yet the biochemical events emanating from prion replication and leading to UPR induction are unknown, and it is unclear how extracellular aggregates can trigger pathology in a subcellular compartment to which they have no direct access.

Prion infection of cerebellar organotypic cultured slices (COCS) has proven to be an extraordinarily faithful and tractable model of prion disease. Prion-infected COCS replicate all salient biochemical, histological, and pathophysiological events which occur during prion infections *in vivo*, including PrP^C^-dependent prion replication [11,12], neuroinflammation with proliferation of microglia and astrogliosis, spongiosis, and neuronal cell loss. In prion-infected COCS, calpain inhibition confers neuroprotection without reducing prion replication, suggesting that calpains are involved in neurotoxicity [13].

We have reported that exposure to antibody-derived anti-PrP ligands (full-length antibodies, F(ab)_1_ fragments thereof, and recombinant single-chain miniantibodies) targeting the globular domain (GD) of PrP^C^ [14] induces rapid cerebellar granular cell (CGC) degeneration in COCS and in live mice. Since this toxic effect was also attenuated by calpain inhibitors [15], we wondered whether the two triggers of PrP-dependent cell death, GDL and prions, might induce similar neurotoxic cascades.

Here we report that antibodies against the flexible tail (FT) of PrP^C^, which prevent GD ligand (GDL) toxicity in COCS [15], also counteracted neurotoxicity in prion-infected COCS, suggesting a role for the FT in both models. Furthermore, GDL treatment and prion infection triggered similar intracellular cascades including PERK activation [9] and reactive oxygen species (ROS) production. Also, a comparative analysis of transcription in prion-infected vs. GDL-exposed COCS showed extensive similarities between these two paradigms of PrP-related toxicity. We conclude that prions and GDL share downstream pathways of toxicity, and that in both instances the FT is the main molecular effector of prion-mediated toxicity.

## Results

### PrP^C^-dependent neurodegeneration of prion-infected and GDL-exposed COCS

Rapid neurotoxicity is elicited in COCS and *in vivo* by several monoclonal antibodies, single-chain variable fragments (scFv), and F(ab)_1_, and F(ab)_2_ fragments directed against the globular domain of PrP^C^ [15]. We collectively termed these reagents “globular domain ligands” (GDL). In all of the experiments described below, we used the POM1 holoantibody (67nM) as a validated paradigm of GDL-associated toxicity. Also, we have previously reported that neurodegeneration and prion replication similarly occur in COCS exposed to the three prion strains, RML, 22L, and 139A [13]. Here, we used RML infection as an extensively characterized paradigm of prion infection. Prion infection of COCS from *tg*a*20* transgenic mice overexpressing PrP^C^ [16] elicited toxicity more rapidly than in wild-type COCS [13], and was used for all experiments except when otherwise indicated. As controls, pooled mouse immunoglobulins (IgG) and non-infectious brain homogenate (NBH) were used.

**Fig 1:**
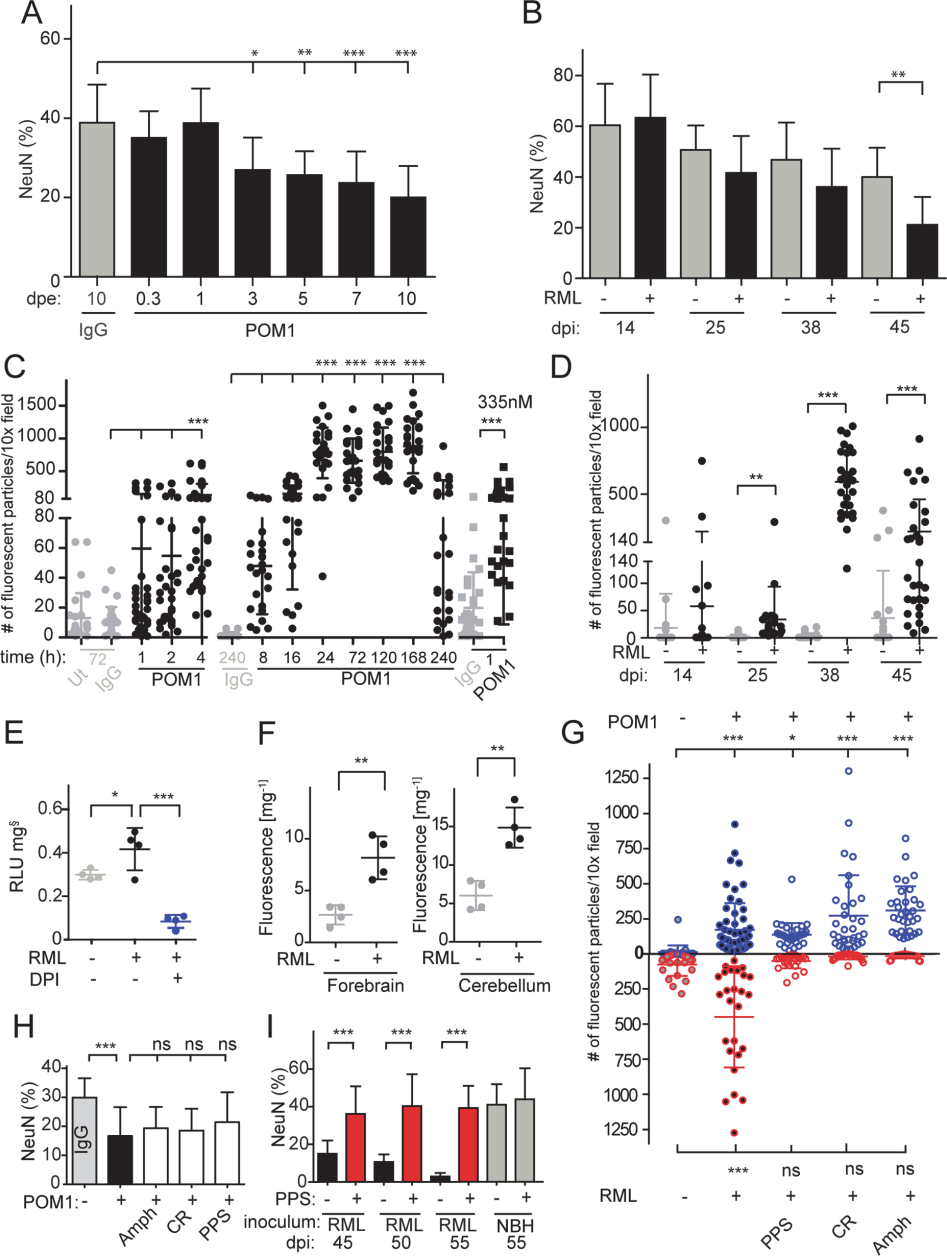
ROS is produced in both prion-infected and GDL-exposed COCS. (**A**) COCS were exposed to POM1 for 8 h-10 days, and neuronal loss was assessed by NeuN^+^ pixel coverage. Neuronal loss was significant at 3 dpe. Untreated slices (ut), or slices exposed to pooled IgG, were used as controls (grey). (**B**) In prion-infected COCS (+) neuronal loss became significant at 45 dpi. Controls (-): exposure to non-infectious brain homogenate. Data were analyzed using a two-tailed t-test; *n* = 9 biological replicates. (**C—D**) COCS were exposed to DHE, and the number of positive fluorescent particles was counted per 10x view field. (**C**) DHE conversion assays were performed at various time points between 1 h and 240 h. Data from three experiments are represented in this graph; *n* = 27 biological replicates. Slices that were untreated or exposed to pooled IgG were used as controls (grey). ROS production was detectable 4 hours after POM1 exposure and after 1 h with a higher POM1 concentration (335 nM). Here and henceforth data are presented as average ± s.d. and were analyzed by one-way ANOVA with Dunnett’s post-hoc test unless otherwise specified (***: *p*<0.001; **p<0.01; *p<0.05; n.s., not significant). Results of IgG vs POM1 at 335 nM were analyzed using a two-tailed t-test. (**D**) COCS were infected with RML (+) or exposed to non-infectious brain homogenate (-). DHE conversion assays were performed at various dpi. ROS production peaked at 38 dpi. Data were analyzed using a two-tailed t-test; *n* = 27 biological replicates. (**E**) RML-infected slices (42 dpi) were harvested and analyzed for ROS production by the lucigenin assay in the presence (blue) or absence (black) of DPI. DPI decreased superoxide-induced lucigenin conversion; *n* = 4 pools of 9 biological replicates. (**F**) DHE conversion was prominent in the forebrains and cerebella of terminally ill scrapie-infected *tg*a*20* mice. Data were analyzed using a two-tailed t-test; *n* = 4 mice. (**G**) The prion replication antagonists, PPS, CR and Amph, suppressed ROS production in prion-infected COCS (lower half), but not in GDL-exposed COCS (upper half); *n* = 27 biological replicates. (**H**) Treatment with PPS, CR, and Amph did not reduce cell death in GDL-treated COCS; *n* = 9 biological replicates. Neuronal viability was assessed here and henceforth by measuring the percentage of NeuN^+^ pixels (ordinate). (**I**) PPS treatment rescued cell death in RML-infected slices for ≥55dpi (red bars). Data were analyzed using a two-tailed t-test; *n* = 9 biological replicates.

First, we compared the progression of neurodegeneration in GDL-exposure vs. prion infection of COCS by measuring the area positive for neuronal-nuclear antigen (NeuN) within the CGC layer, and by counting cells stained by propidium iodide (PI). The NeuN^+^ area was used to estimate COCS viability, while the density of PI^+^ cells correlated with the intensity of ongoing damage. A previously published time-course experiment [15] was repeated including additional time points. PI^+^ cells peaked at 3 days post-exposure (dpe) (S1A Fig.) and decreased around 7–10 dpe in GDL-treated COCS. Also, significant loss of NeuN^+^ granule cells was detectable at 3 dpe (Fig. 1A). In prion-infected COCS, we observed a peak of PI^+^ cells at 38 days post infection (dpi) (S1B Fig.) and significant neuronal cell loss at 45 dpi (Fig. 1B).

### GDL and prions induce pathogenic cascades that converge on ROS induction

ROS, particularly superoxide, are causally involved in GDL toxicity [15]. We therefore asked whether prion infection resulted in ROS production, and whether ROS scavenging might be beneficial. We measured ROS production in live GDL-treated and RML-infected COCS by fluorescent recording of dihydroethidium (DHE) oxidation products [17]. GDL-treated COCS were treated with DHE at various time points between 1 h and 10 days after POM1 exposure (Fig. 1C). Enhanced fluorescence from DHE oxidation products was observed at 4 h (67 nM). Exposure to a fivefold higher POM1 concentration (335nM) resulted in toxicity even after 1 h. Significantly increased fluorescence was observed in prion-infected COCS (Fig. 1D, RML “+”) starting at 25 dpi and reached a peak at 38 dpi, but not in COCS exposed to non-infectious brain homogenate (RML “-”).

Consistently with what we found in GDL-exposed *tg*a*20* COCS, we observed significant ROS production, measured by DHE incorporation, in GDL-exposed COCS from wild-type (Bl/6) mice at 7 and 14 dpe (S2A Fig.). This result provides further validation for our view that prion related pathologies show very similar characteristics in wild-type and *tg*a*20*-derived tissues.

ROS generation was also measured by lucigenin conversion, which detects superoxide anion radicals [18]. COCS exposed to GDL displayed increased lucigenin conversion [15], which was quenched by diphenylene iodonium (DPI), an inhibitor of ROS-producing electron transporters including NADPH oxidases (NOX) [15]. Similarly, we observed elevated lucigenin conversion in prion-infected COCS at 42 dpi, indicating a strong increase in superoxide production. Furthermore, addition of DPI quenched ROS production in prion-infected COCS (Fig. 1E).

We also measured ROS production *in vivo* using DHE. Terminally sick RML-infected mice were injected intraperitoneally with DHE, and DHE oxidation products were detected in brain homogenates. Forebrains and cerebella of prion-infected mice showed higher levels of fluorescence than NBH-inoculated control mice (Fig. 1F).

If the superoxide burst in prion-infected COCS is a direct consequence of prion infection, interference with prion replication should reduce ROS production. We therefore subjected prion-infected COCS to a panel of compounds that had previously been found to antagonize prion replication, including pentosan polysulfate (PPS), congo red (CR), and amphotericin B (Amph). Prion-induced ROS production was reversed by treatment with PPS, CR, and Amph (Fig. 1G lower half). Hence, ROS production is a general feature of prion toxicity downstream of prion replication.

PPS, CR, and Amph may be effective because they intercalate with prions, or because they activate neuroprotective pathways independently of their interactions with PrP^Sc^. We therefore tested the effects of PPS, CR, and Amph on GDL-treated COCS. We found that ROS production was not reduced (Fig. 1G, upper half) and neurodegeneration was not prevented (Fig. 1H), whereas PPS, CR, Amph counteracted neurotoxicity in prion-infected COCS [13], with PPS being protective for at least 55 dpi (Fig. 1I). We conclude that the prionostatic properties of these compounds, rather than any off-target effects, were indeed the proximal reason for ROS suppression.

### ROS scavengers protect against prions and GDL in vitro and in vivo

**Fig 2:**
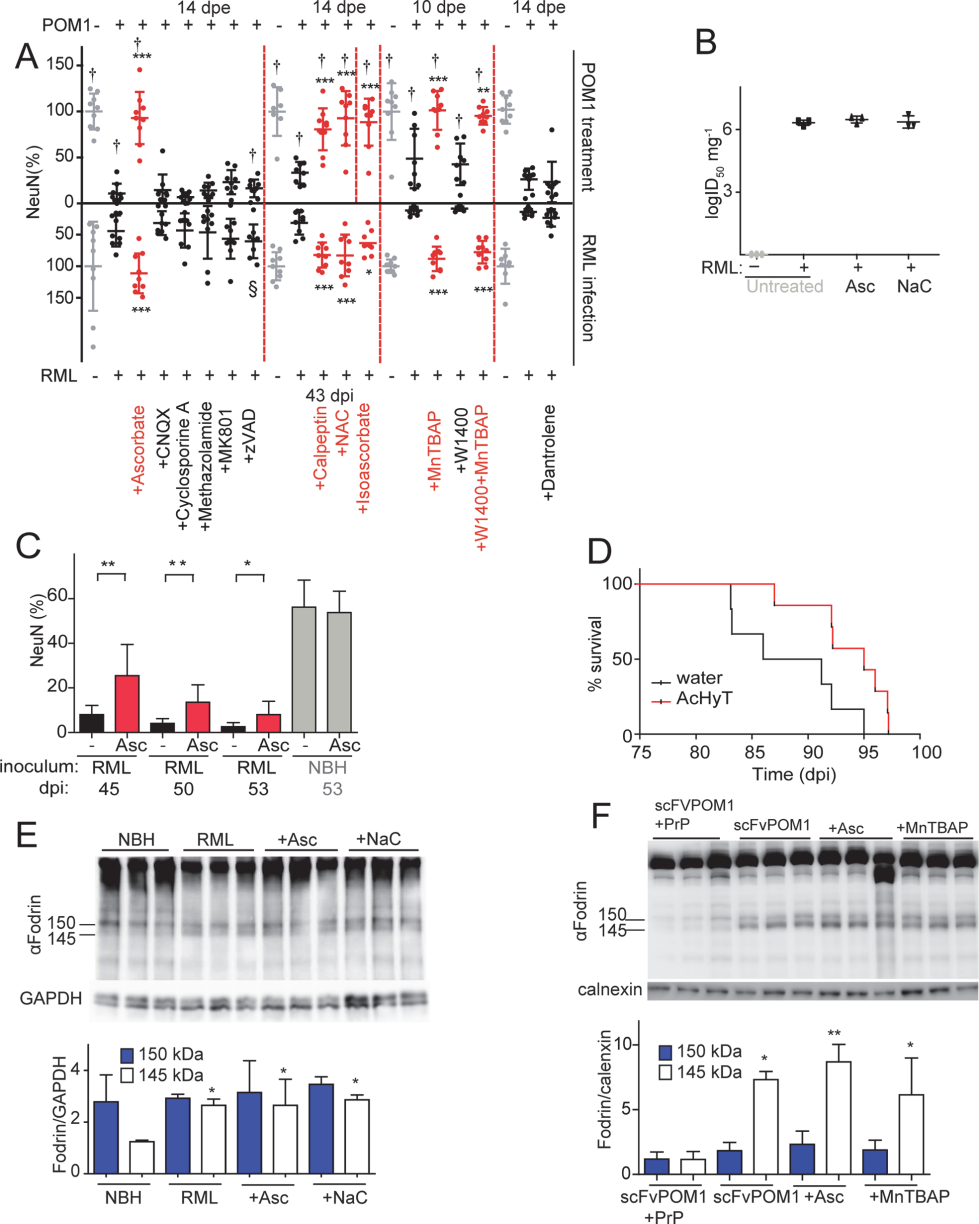
ROS scavengers and calpain inhibitors are beneficial to prion-infected COCS and mice. (**A**) Lower graph: COCS were inoculated with RML or NBH, cultured for 21 dpi, and then treated with various compounds for 22 days. Upper graph: COCS were cultured for 14 days, incubated with POM1 and treated with the same compounds for 10–14 days. Viability (NeuN) was normalized to COCS exposed to IgG and to non-infectious brain homogenate (upper and lower gray dots, respectively). Calpain inhibitors (Calpeptin), antioxidants (Ascorbate, Isoascorbate, NaC), and superoxide dismutase mimetics (MnTBAP) were neuroprotective in both paradigms (red dots), whereas inhibition of AMPA, kainate receptors (CNQX), NMDA receptors (MK801), a mitochondrial membrane permeability transition pore (methazolamide), caspases (ZVAD), and of ER calcium stores (Dantrolene), (black dots) was ineffective; *n* = 9 biological replicates. The effects of compounds labeled with “†” on POM1-exposed COCS and zVAD labeled with “§” on RML-infected COCS were reported previously [15], [13] and are reproduced here for convenience. (**B**) COCS were exposed to NBH (-) or to RML (+) as in (A), and harvested at 39 dpi (*n* = 3). Prion titers were determined by SCEPA. No reduction in prion infectivity was observed in COCS treated with ascorbate (Asc) or NaC (*n* = 3 biological replicates). (**C**) Prion-infected COCS were treated with Asc. Protection was discernible for ≥53 dpi (red bars). Data were analyzed using a two-tailed t-test; *n* = 9 biological replicates. (**D**) Survival curves of prion-infected *tg*a*20* mice treated with AcHyT (median 95dpi) or vehicle (drinking water; median 88.5dpi). AcHyT treatment increased the incubation time of prion-infected mice (p = 0.0287). Results were analyzed using a Mantel-Cox log-rank test; *n* = 6 untreated mice and *n* = 7 AcHyT-treated mice. (**E**) COCS were treated with antioxidants from 21 dpi onwards (*n* = 3) and harvested at 40 dpi. Cleaved α-fodrin bands (145 and 150 kDa) were quantified and normalized to GAPDH (right). None of the antioxidants suppressed the prion-induced enhancement of α-fodrin cleavage; *n* = 3 biological replicates. (**F**) COCS were cultured for 14 days, treated with scFvPOM1 (400nM) or scFvPOM1 preincubated with recombinant PrP (1200nM) in presence of ascorbate, and harvested at 3 dpe. Cleaved α-fodrin bands (145 and 150 kDa) were quantified and normalized to calnexin (right). Ascorbate did not suppress the GDL induced enhancement of α-fodrin cleavage; *n* = 3 biological replicates.

Analogously to what we observed in GDL-exposed COCS, the ROS scavengers ascorbate and N-acetyl cysteine (NaC) completely prevented prion neurotoxicity in COCS (Fig. 2A), although neither compound affected prion titers (Fig. 2B). Furthermore, ascorbate did not affect PrP^Sc^ accumulation, total PrP levels, or processing of PrP^C^ into the C1 fragment in prion-infected COCS. Only the C2 fragment was decreased (S3A–S3D Fig.).

As previously shown for GDL toxicity, the membrane-impermeable antioxidant isoascorbate and the superoxide dismutase mimetic MnTBAP conferred protection against prion toxicity, suggesting that the relevant ROS species are extracellular in both instances (Fig. 2A). In contrast, the nitric oxide synthase inhibitor 1400W was ineffective in both prion-infected (Fig. 2A) and GDL-exposed COCS [15]. These data indicate that the ROS moiety instrumental to prion-induced neurodegeneration is superoxide, rather than nitric oxide, in both models.

We then investigated whether ascorbate would be neuroprotective over protracted periods of time. RML-infected COCS were treated with ascorbate and harvested at various times between 45–53 dpi. Remarkably, ascorbate significantly reduced neurodegeneration of RML-infected COCS for ≥53 dpi (Fig. 2C). Finally, we asked whether antioxidants might be protective against prion-induced neurotoxicity *in vivo*. For this, we administered the enterically activated antioxidant, acetylated hydroxy tyrosol (AcHyT, 2g l^-1^ added to drinking water) [19], to *tg*a*20* transgenic mice. AcHyT was previously shown to block the toxicity of GDL *in vivo* [15]. *Tg*a*20* mice pretreated with AcHyT for 7 days were intracerebrally infected with 22L prions (30μl, diluted 10^–2^) and treatment with AcHyT was continued until mice reached the criteria for termination of the experiment. Treated animals showed a modest, but significant, life extension (Fig. 2D). Hence, AcHyT is protective *in vivo* against the toxicity of both prions and GDL.

We have previously shown that calpain inhibitors, but not caspase inhibitors, prevent cell death in GDL-exposed [15] and RML-infected COCS [13] (Fig. 2A). In order to test whether ROS signaling occurs upstream of calpain activation, we studied the effects of antioxidants on the catabolism of fodrin, which is specifically cleaved by calpains into fragments of 145 and 150 KDa. This cleavage was blocked by calpain inhibition [13] yet was unaffected by antioxidant treatment in both RML-infected and GDL-exposed COCS, indicating that ROS production is triggered by events that are dependent on (“downstream” of) calpain activation (Fig. 2E, F). This hierarchical sequence may not be unique to PrP-related toxicity, and other calpain activators may plausibly also induce ROS.

### Anti-excitotoxic compounds are ineffective against both GDL and prion-induced neurotoxicity

Excitotoxicity is a potent ROS inducer [17], and PrP^C^ can modulate NMDA and voltage-gated calcium channels [20,21]. We therefore investigated if inhibitors of NMDA and AMPA/kainate ionotropic glutamate receptors, or of a mitochondrial membrane permeability transition pore, could protect COCS against GDL or prion neurotoxicity. However, none of the inhibitors were protective in either model (Fig. 2A). Also, inhibiting ryanodine receptor-mediated calcium release from the endoplasmic reticulum (ER) with Dantrolene, was not protective (Fig. 2A). None of the tested compounds were inherently toxic, as the viability of IgG-treated or NBH- exposed *tg*a*20* COCS were unaffected (S4 Fig.).

### Binding of the flexible tail of PrP^C^ delays neurodegeneration in prion-infected COCS

**Fig 3:**
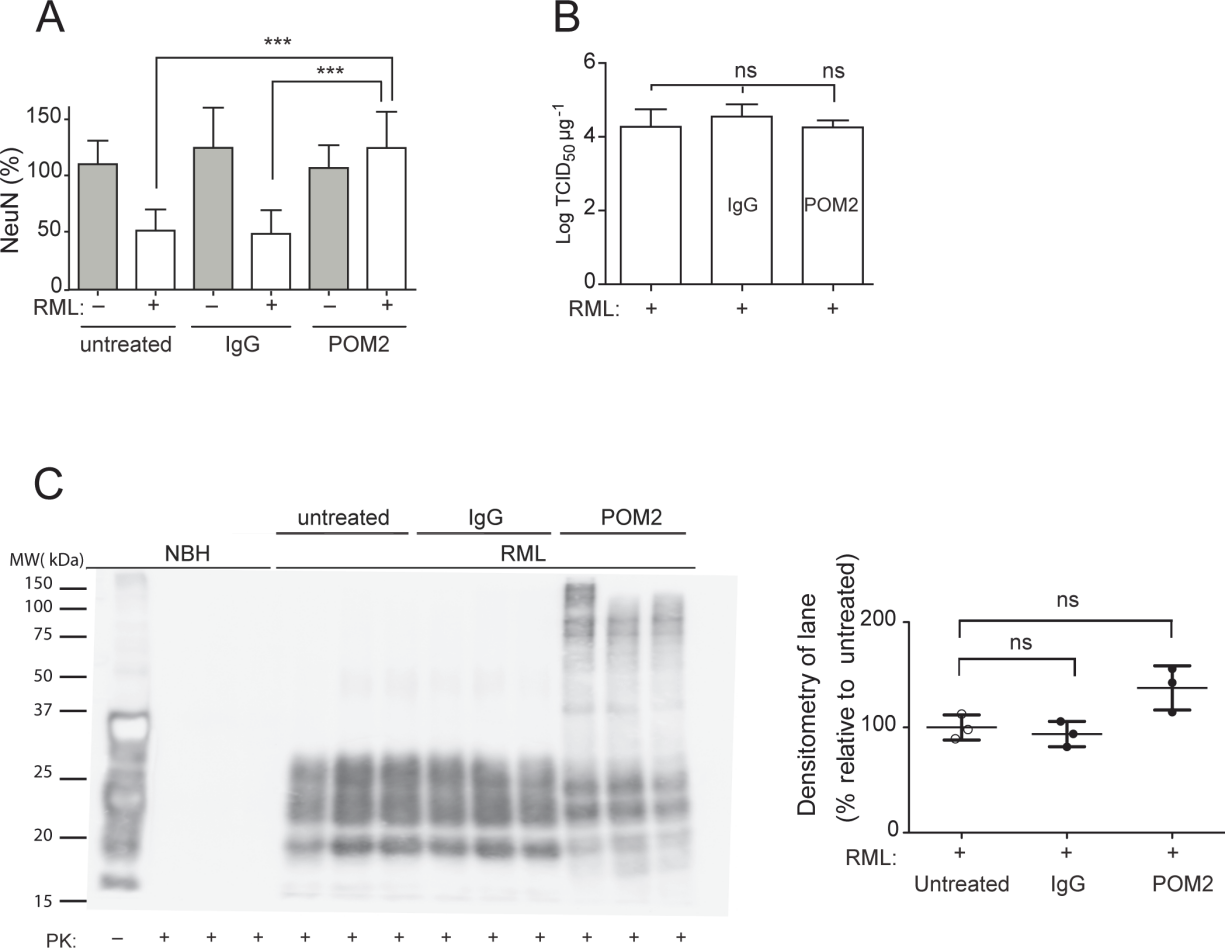
anti-FT antibody POM2 counteracts prion-induced neurotoxicity in COCS. (**A**) COCS prepared from *tg*a*20* mice were exposed to NBH (gray) or infected with RML prions (white), treated with pooled IgG or POM2 (335nM), and stained for NeuN at 44dpi. POM2 treatment afforded significant neuroprotection against prion infection; *n* = 9 biological replicates. (**B**) Prion titers of slice homogenates treated as in (A) were determined by SCEPA. POM2 treatment did not significantly reduce infectivity titers in prion-infected COCS. Data points represent the log of the median infectivity dose per mg of tissue culture (TCID_50_ mg^-1^) ± s.d.; *n* = 4 biological replicates. (**C**) COCS homogenates were treated as in (A), digested with proteinase K, and probed with the antibody POM1 for PrP^Sc^. While the PrP^Sc^ bands between 18–30 kDa were somewhat decreased by POM2 treatment, there was a concomitant increase in higher-molecular PrP-immunoreactive moieties presumably representing SDS-resistant oligomers of PrP^Sc^. Because of the different electrophoretic patterns of POM2-treated samples, we have opted to perform a quantification of the entire lane (right panel). POM2 treatment did not decrease the load of PK-resistant PrP^Sc^; *n* = 3 biological replicates.

High-affinity ligands to the FT of PrP^C^ such as the POM2 antibody [14] are not only innocuous, but counteract the toxicity of GDL. Moreover, interstitial FT deletions prevent GDL toxicity *in vitro* and *in vivo*, indicating that the FT is required to execute GDL toxicity [15]. To determine whether the FT also mediates toxicity in prion infection, we treated prion-infected COCS with the POM2 antibody, which recognizes the octapeptide repeats of the FT. POM2 prevented prion-mediated neurodegeneration in *tg*a*20* COCS, whereas equimolar amounts of IgG had no beneficial effect (Fig. 3A). We determined prion titers by the scrapie cell-assay in end-point format (SCEPA; [22,23], Fig. 3B), which measures the minimal concentration that still can infect the cells and is currently the most precise measurement of infectivity *in vitro*. Crucially, prion titers were not significantly affected. This finding disproves the possibility that neuroprotection was caused by reduced infectivity, and suggests that POM2 acted specifically on prion neurotoxicity by interfering with events triggered by the encounter of prions with their target cells.

Western blots of PK-digested samples showed that POM2 treatment led to the appearance of PrP-immunoreactive higher-molecular weight bands (Fig. 3C), possibly representing SDS-stable PrP^Sc^ oligomers concomitant with reduced immunoreactivity in the 27–30 kDa range. The total PK-resistant PrP immunoreactivity was determined by densitometric quantification of the entire lane, and was found to be similar to that of samples that had not been exposed to POM2. We conclude that POM2 induced a shift in the distribution of PrP^Sc^ moieties without affecting its overall quantity. This interpretation is congruent with the results of prion titer determinations (Fig. 3B).

### The ER stress pathway is involved in anti-GD antibody and prion-induced toxicity

**Fig 4:**
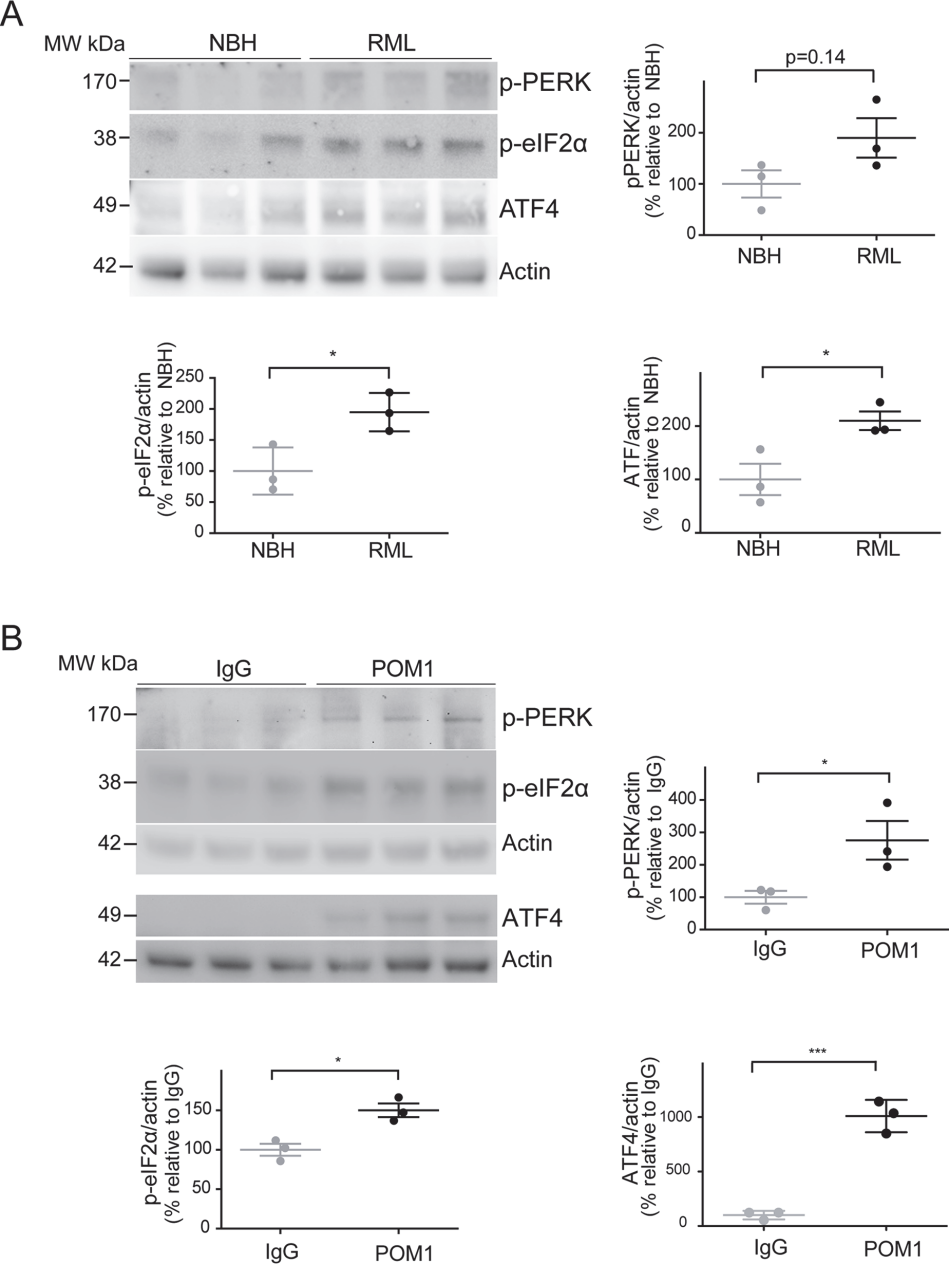
ER stress is detected in both prion-infected and GDL-exposed COCS. (**A**) *Tg*a*20* COCS (prion-infected or NBH-exposed) were harvested at 42 dpi, and probed for p-PERK, p-eIF2α, and ATF4 by western blot. Densitometry (normalized to actin) showed a trend towards increased p-PERK (*p* = 0.14) and significantly elevated p-eIF2α and ATF4 after prion infection. (**B**) COCS were cultured for 14 days, treated with POM1 or IgG, harvested at 3 dpe, and probed for p-PERK, p-eIF2α and ATF4 by western blot. Densitometry (normalized to actin) revealed increased p-PERK, p-eIF2α, and ATF4. Densitometry data were analyzed using a two-tailed t-test; *n* = 3 biological replicates.

Since ER stress has previously been shown to be involved in prion disease [9,10], we examined the levels of phosphorylated PERK (p-PERK), phosphorylated eIF2α (p-eIF2α), and ATF4 in both paradigms of PrP^C^-dependent neurotoxicity. We found a trend towards increased levels of p-PERK, as well as significantly increased p-eIF2α and ATF4 in RML-infected COCS at 42 dpi (Fig. 4A), confirming the activation of the unfolded protein response in prion infections. Surprisingly, we found that POM1-exposed COCS also showed increased p-PERK, p-eIF2α, and ATF4 at 3 dpe (Fig. 4B).

To further explore this phenomenon, COCS prepared from wild-type mice were exposed to POM1 for 7 and 14 days (S2B–S2C Fig.). At 7 dpe there was a trend towards increased p-PERK and p-eIF2α levels, whereas ATF4 was unchanged. At 14dpe we found significantly increased levels of p-eIF2α and ATF4, suggesting again an involvement of the PERK pathway as observed in the *tg*a*20* COCS. This suggests that signals emanating from GDLs can propagate to the ER and initiate a response similar to that seen in prion infections.

### Prion-infected and GDL-treated COCS share transcriptional changes

**Fig 5:**
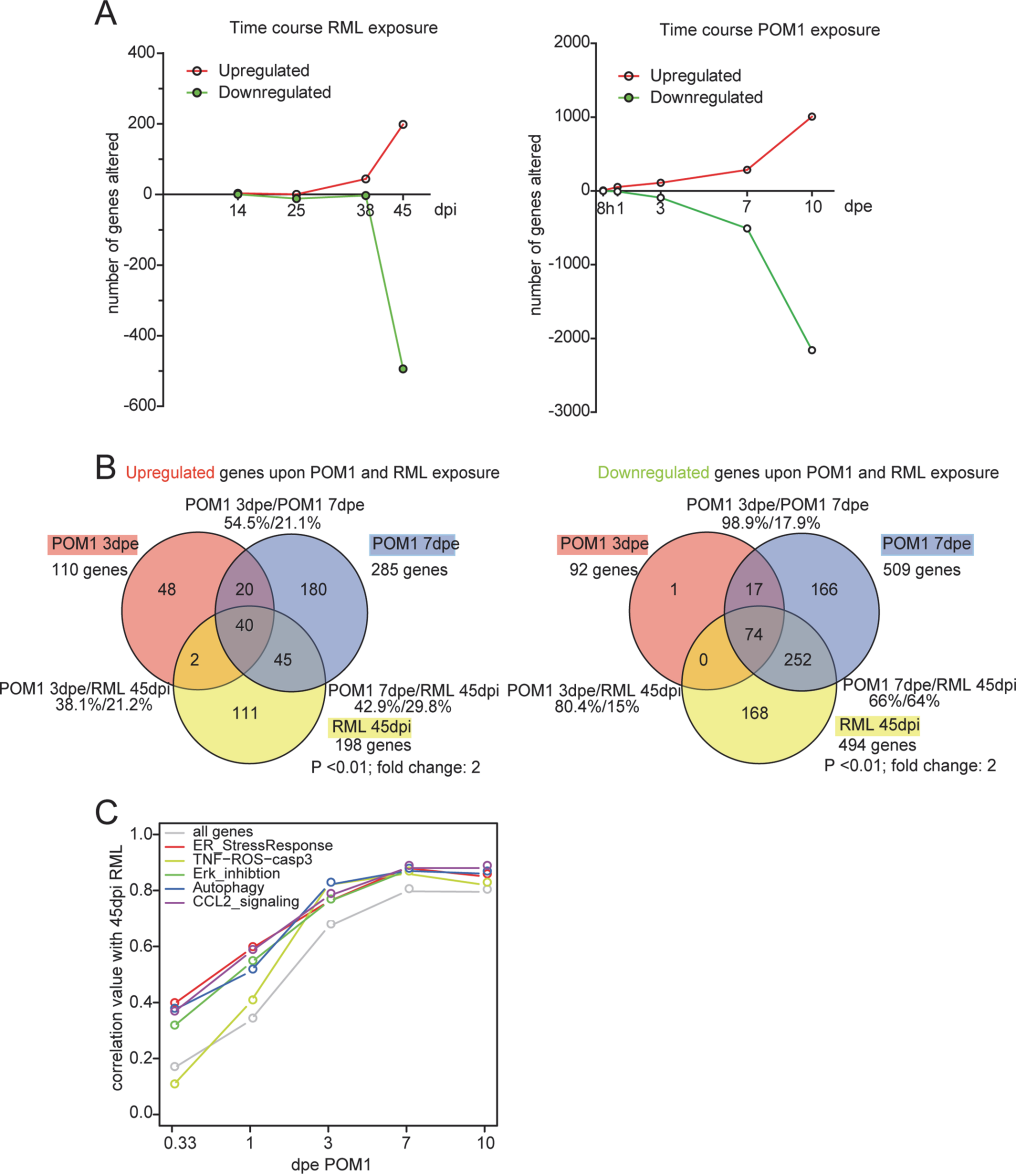
The transcriptome of RML-infected and POM1-treated COCS. (**A**) The number of genes upregulated or downregulated at various time points after RML infection (left) or exposure to POM1 (right). Upregulation or downregulation was defined as >2-fold change over NBH-exposed (left) or pooled-IgG exposed (right) COCS at the same time point. In both paradigms, the total number of differentially expressed genes increased over time. (**B**) Overlap of upregulated (left) and downregulated (right) genes in RML-infected vs. POM1-exposed COCS. The two paradigms shared 80% of downregulated and 38% of upregulated genes when comparing 3dpe GDL and 45dpi RML. (**C**) Correlation coefficients (y-axis) of all genes and genes involved in specific signaling pathways between POM1 exposure at different time points (x-axis) and RML exposure at 45 dpi.

The transcriptional changes occurring in COCS infected with prions and exposed to GDL were studied by microarray hybridization. Genes were considered to be differentially expressed if they exhibited a fold change of ≥ 2 (*p* value < 0.01) between RML and NBH (prion infection paradigm) or between POM1 and IgG (GDL exposure paradigm). Upregulated and downregulated genes were compared at various time points (Fig. 5A; S1–S2 Tables). To account for the different velocity of neurodegeneration between the two models, we compared transcriptional profiles at the time at which NeuN staining loss became significant (3 dpe for GDL vs. 45 dpi for prion infection). The largest overlap of transcriptionally altered genes was found when GDL-treated COCS at 3 dpe were compared to prion-infected COCS at 45 dpi. At these time points, COCS shared 38% of all upregulated genes (Fig. 5B left; S3 Table) and 80% of all downregulated genes (Fig. 5B right). At the peak of ongoing cell death in both models (3 dpe for GDL vs. 38 dpi for prion infection; S1A–S1B Fig., measured by PI incorporation), we found that 38.4% (15/39) of upregulated genes were identical. Only one of these fifteen genes, *Fos*b, has been annotated as possibly involved in the signaling initiated by activated ROS [24]. The remaining 14 genes have been assigned to various cellular pathways, but we failed to identify an overrepresentation of any specific pathway.

The analysis of individual genes, followed by the compilation of a list of candidates using arbitrary cut-off criteria (typically fold-change and *p* values) may not reveal biologically important effects on pathways. For example, a modest yet concerted increase in the activity of several genes feeding into the same pathway may be more consequential that a strong increase of a single member gene. We therefore set out to evaluate microarray data as predefined gene sets that could be assigned to certain pathways. Gene set enrichment analysis (GSEA) is an important approach to the analysis of coordinate expression changes at a pathway level [25]. Specifically, we applied this method to our microarray data in order to specifically investigate whether the genes constituting the TNF-ROS-CASP3 pathway are significantly regulated in a coordinated manner in POM1 and RML-exposed COCS. Indeed, we found using GSEA, that the TNF-ROS-CASP3 pathway indeed was significantly regulated in both POM1-exposed COCS after 3d (p = 0.037) and RML-exposed COCS after 38d (p = 0.03) and after 45d (p = 0.026). This result shows that in both paradigms genes belonging to the same ROS-dependent pathway are activated upon exposure. Only 3 genes were downregulated at 38 dpi, one of which was downregulated at 3 dpe in GDL-exposed COCS.

The top 40 upregulated pathways at each time point were identified using GeneGo MetaCore software. When comparing POM1 at 3 dpe to prion infection at 45dpi, there was an overlap of 19 pathways (47.5%), while 9 out of the top 10 active pathways at 45dpi RML were present in the top 40 of POM1 7 dpe (S4 Table).

When genes in POM1-treated COCS at different time points were compared with 45 dpi prion-infected COCS, the correlation of genes increased with time and reached a plateau at 7 dpe POM1, with a correlation coefficient close to 0.8 (Fig. 5C). We then examined the involvement of pathways from the GeneGo MetaCore database that had been described to be activated upon prion infection, such as the ER stress response [9], ERK inhibition [26,27], autophagy [28], CCL2 signaling [29], and TNF-ROS-casp3 [29,30]. These pathways were activated in POM1-treated COCS in a pattern that strongly correlated with RML-infected COCS at 45dpi (correlation value between 0.8–0.9). Scatter plots and heat maps of the genes involved in the five signaling pathways (S5A–S5E Fig.; S6 Fig.) support this view.

To validate the regulation of genes in the microarray data, we performed nanostring analysis for 40 genes on the same RNA preparation that was used in the microarray analysis. Differential expression of the selected genes at various time points in prion-infected and GDL-exposed COCS (S5 Table) confirmed the results of the microarray analysis.

## Discussion

Using multiple paradigms in organotypic cultures and *in vivo*, we show that the toxic antibody POM1 induces largely overlapping pathogenetic cascades as *bona fide* prion infections. Not only were all strategies preventing GDL-induced neurodegeneration (such as calpain inhibition, ROS scavenging and FT binding) found to be neuroprotective against prions, but compounds neuroprotective against other kinds of insult (such as caspase inhibitors and glutamate antagonists) were ineffective against both GDL and prions. Moreover, the results of transcriptomic analyses are compatible with the contention of a large overlap in the downstream effectors of both pathways. Besides highlighting the commonalities between GDL and prion-related neurodegeneration (S7 Fig.), these observations set both conditions apart from other types of neurodegenerative conditions.

Treatment of prion-infected COCS with antioxidants did not interfere with the aggregation of PK-resistant material, as was previously shown for calpain inhibitors [13]. This adds to the evidence that ROS scavengers and calpain inhibitors mitigate toxicity by interfering with events triggered by prion replication. A plausible model of pathogenesis predicates that toxicity is triggered by binding of either GDL or PrP^Sc^ to the globular domain of PrP^C^. Since inhibitors of prion replication decreased ROS production, but did not protect from GDL toxicity and did not reduce ROS production (Fig. 1G), we conclude that ROS production is downstream of both prion replication and GDL binding.

The modest therapeutic effect of the antioxidative therapy with AcHyt *in vivo* is not unexpected, since their involvement occurs downstream of both prion replication and calpain activation, and suggests the existence of additional pathways of toxicity that remain operational even after scavenging ROS.

How can GDL execute such a faithful molecular mimicry of prion infection? We favor the hypothesis that GDL and PrP^Sc^ share the same docking site on cellular PrP^C^. Engagement of the latter site enacts a long-range allosteric transition of the FT, which in turn triggers the toxic cascade. The above scenario cannot be tested directly because of the technological barriers still hampering structural studies of PrP^Sc^, yet it is at least compatible with the structure of the POM1:PrP^C^ complex, as determined by X-ray crystallography [31,32].

Solforosi et al [33] claimed that anti-PrP antibodies induced toxicity by crosslinking PrP^C^, as F(ab)_1_ fragments were innocuous in their study. However the monovalent scFv and F(ab)_1_ fragments of antibody POM1 lead to toxicity *in vitro* and *in vivo* [15]. This refutes clustering of PrP^C^ as a cause of toxicity in the present study.

Thus far, quests for anti-prion therapeutics have been rare and mostly unsuccessful. Two crucial reasons are the hazards associated with prion infectivity and the dearth of rapid, validated models of prion-induced toxicity. The validation of GDL as prion mimetics will help identify novel nodes in the pathogenetic cascades leading to neurodegeneration, and it is likely that some of these nodes may represent druggable targets.

We also identified differences between the pathogenesis of GDL exposure and that of prion infections. Firstly and most glaringly, the kinetics of GDL-induced neurodegeneration (days) is much faster than that of experimental prion infections (months). Secondly, although GDL function as a prion mimetic, they differ from bona fide prions by not inducing the classical misfolding and aggregation of PrP, by failing to induce deposition of protease-resistant PrP, and most crucially by being non-infectious. Thirdly, compounds that interfere with prion replication, such as pentosan polysulfate, do not alter the toxicity caused by GDL. All of these observations are compatible with the interpretation that GDL, while not leading to the generation of PrP^Sc^, trigger the same signaling pathway as prion infections.

The different speed of disease development may be taken to suggest that GDL exposure and prion infections are fundamentally dissimilar. In our view, however, this does not contradict the hypothesis that these two models share pathogenetic pathways. Prion infection is initiated by trace amounts of prions within brain homogenates, with the PrP^Sc^ concentration only gradually increasing upon infection of a progressively larger numbers of host cells.

The prion isolate used for a standard slice culture infection contains 7.9*10^6^ ID_50_ in 10 μl [12]. Antibodies in slice culture experiments were used at 67 nM in 1 ml of medium, which corresponds to 4.0*10^13^ molecules. We conclude that, in the case of the antibodies, the exposure to the bioactive principle exceeds that of prions by ca. 7 logs. This calculation is conservative since it disregards that prion inocula were removed after exposure, and that large prion aggregates are unlikely to efficiently penetrate tissues. Hence the bioactive principle in antibody preparations exceeds that in prion infections by several orders of magnitude, which yields—in our view—a highly plausible explanation for the difference in the kinetics of neurodegeneration.

Finally, recent evidence suggested the involvement of the unfolded protein response in prion-induced cell death *in vivo* [9]. Here, we confirm its involvement in RML and GDL-induced cell death in COCS. All of the above suggests that GDL-induced neurodegeneration represents a phenocopy of *bona fide* prion infections. If this conjecture is correct, targeted manipulations of the FT may be beneficial against neurotoxicity in prion infections. Indeed, we found that binding of the FT by antibodies was neuroprotective to prion-infected slice cultures, yet did not appreciably reduce prion titers—indicating selective suppression of the cytotoxic events downstream of prion replication. Therefore, binding of the FT could modify the course of the disease by uncoupling prion replication from prion toxicity. This hypothesis remains to be validated.

Since the GDL-induced toxicity model closely mimics multiple aspects of prion-induced neurotoxicity of prion-induced neurotoxicity, it seems reasonable to utilize GDLs for phenotypic screens aimed at identifying potential antiprion therapeutics. While confirmatory counterscreens will still require proof of efficacy against infectious prions, we posit that GDL toxicity may form the basis of convenient high-throughput and non-biohazardous assays of chemical and biological libraries.

## Materials and Methods

### Chemicals

All compounds were purchased from Sigma/Aldrich unless otherwise stated.

### Mice

*Prnp* ^o/o^;*tg*a*20* ^+/+^ (*tg*a*20*) mice were on a mixed 129Sv/BL6 background [16,34]. C57BL/6 mice were used as a wild-type mouse strain.

### *In vivo* experiments

10-week old *tg*a*20* mice were administered acetylated hydroxy tyrosol in drinking water ad libitum (2 g l^-1^ with an approximate intake of 8 ml daily). After 7 days of treatment, mice were anesthetized and intracerebrally inoculated with the 22L prion strain (30μl of 1% homogenate into the temporal cortex). Prion-inoculated animals were examined every second day and euthanized upon reaching pre-specified criteria for the terminal stage of disease. All prion-inoculated mice developed typical signs of scrapie and prion infection was confirmed in all cases by western blotting for protease-resistant PrP^Sc^ with the anti-PrP antibody POM1 (S8 Fig.) [14].

### Organotypic brain culture preparation

Cultured organotypic cerebellar slices were prepared as previously described [11]. Briefly, cerebella from 10–12 day old pups were cut into 350 μm sections and kept in Gey’s balanced salt solution (GBSS) (NaCl 8 g l^–1^, KCl 0.37 g l^–1^, Na_2_HPO_4_ 0.12 g l^–1^, CaCl_2_ 2H_2_O 0.22 g l^–1^, KH_2_PO_4_ 0.09 g l^–1^, MgSO_4_ 7H_2_O 0.07 g l^–1^, MgCl_2_ 6H_2_O 0.210 g l^–1^, NaHCO_3_ 0.227 g l^–1^) supplemented with the glutamate receptor antagonist kynurenic acid (1 mM) at 4°C. Six to ten slices were then plated per Millicell-CM Biopore PTFE membrane insert (Millipore) and residual buffer was removed before placing the inserts into a cell culture plate containing “slice-culture medium” (50% vol/vol MEM, 25% vol/vol basal medium Eagle and 25% vol/vol horse serum supplemented with 0.65% glucose (w/vol), penicillin/streptomycin and glutamax (Invitrogen)). Culture medium was exchanged thrice weekly and tissue cultures were kept in a humidified cell culture incubator set to 37°C with 5% CO_2_.

### Antibody treatment and prion inoculation of COCS

Antibody treatment and prion inoculations were performed as previously described [11,15,35]. Briefly, for antibody experiments, POM1 was spiked into the medium 10–14 days post-culturing, a time point at which COCS had recovered from acute phenomena associated with tissue dissection. Fresh POM1 was provided at every medium change. Cultures were harvested for biochemical analyses or fixed for immunocytochemical analyses at different time points.

For prion experiments, immediately after dissection, free-floating sections were incubated with infectious brain homogenates for 1 h at 4°C. Sections were then washed twice in 6 ml GBSSK, and 6–10 slices were transferred onto a 6-well Millicell-CM Biopore PTFE membrane insert (Millipore). Residual buffer was removed and inserts were placed into a 6-well culture plate and incubated in standard slice culture medium. POM2 treatment (335nM) was initiated after plating and re-supplied at every medium exchange.

### Pharmacological treatment of COCS

For antibody experiments, drug treatment was initiated at the time of antibody addition (10–14 days post-culturing), whereas for prion experiments, drug treatment was started at 21 dpi when PrP^Sc^ is detectable in the cultures. Drugs were re-supplied at every medium change. As a control, the toxicity of each compound was tested in parallel in IgG-treated slices and NBH-inoculated slices. The drugs and concentrations used were (+)-5-methyl-10,11-dihydro-5H-dibenzo[a,d] cyclohepten-5,10-imine maleate (MK-801, 20 μM), 6-cyano-7-nitroquinoxaline-2,3-dione (CNQX, 20 μM), cyclosporine A (1 μM), ascorbate (1.5 mM), isoascorbate (1.5mM), N-(3-methyl-5-sulfamoyl-1,3,4-tiadiazol-2-ylidine)acetamide (methazolamide, 10 μM), MnTBAP (100 μM), benzyloxycarbonyl-Val-Ala-Asp (OMe) fluoromethylketone (zVAD-fmk, 40 μM), diphenyleneiodonium chloride (DPI, 5 μM), N-([3-(Aminomethyl)phenyl]methyl)- ethanimidamide dihydrochloride (1400W, 20 μM), N-benzyloxycarbonyl-L-leucylnorleucinal (calpeptin, 20 μM), N-acetylcystein (NaC, 1 mM), (2S,3S)-trans-epoxysuccinyl-L-leucylamido-3-methylbutane ethyl ester (E64d, 15 mM, Bachem), 1-[(5-(p-Nitrophenyl)furfurylidene)amino]-hydantoin sodium salt (Dantrolene, 10 μM). A summary table of the used tool compounds and their biological targets are reported in S6 Table. The drugs and concentrations used for anti-prion compounds were pentosan polysulphate (PPS, 300 ng ml^-1^, generously provided by Bene Pharmachem), Congo red (1 mg ml^-1^), and amphotericin B (4.5 mg ml^-1^).

### Protein analysis

Inserts containing the slices were transferred to new plates containing PBS for washing (twice) and tissue was then scraped off the membrane using 10 μl per slice of lysis buffer (0.5% sodium deoxycholate (DOC), 0.5% Nonidet P-40 (NP-40) supplemented with complete mini protease inhibitor cocktail (Roche) and PhosphoStop (Roche) in PBS). The harvested tissue was homogenized by trituration using a 30G syringe and protein concentrations were measured using the bicinchoninic acid assay (Pierce). Samples were mixed with loading buffer (NuPAGE, Invitrogen) and heated at 95°C for 5 min. Equal volumes were loaded (10 μg proteins per lane) and separated on a 12% Bis-Tris polyacrylamide gel or for higher molecular weight proteins, on a 4–12% gradient gel (NuPAGE, Invitrogen), and transferred onto a nitrocellulose membrane. These membranes were blocked with 5% w/vol Topblock (Fluka) in TBS-T (Tris-buffered saline supplemented with Tween20 (150 mM NaCl, 10 mM Tris HCl, 0.05% Tween 20 (vol/vol)) for 1 h and incubated with primary antibodies diluted in 1% Topblock in TBS-T at 4°C overnight. After 4 washes of 15 minutes each with TBS-T, membranes were incubated with secondary antibody diluted in 1% Top Block in TBS-T for 1 h at RT. Primary mouse monoclonal antibodies used were: POM1 mouse IgG_1_ antibody raised against PrP^C^ (anti-PrP^C^; 200 ng ml^–1^), anti-α-fodrin (AA6, 100 ng ml^-1^, Millipore), anti-GAPDH (200 ng ml^-1^, Millipore), anti-actin (200 ng ml^-1^, Chemicon) and anti-calnexin (1:3000, Enzo Life Sciences). Secondary antibodies were horseradish peroxidase (HRP)-conjugated rabbit anti–mouse IgG_1_ (1:10,000, Zymed), and goat anti–rabbit IgG_1_ (1:10,000, Zymed). The following rabbit monoclonal antibodies were used: anti-phospho PERK (Cell Signal 3179S), anti-PERK (Cell Signal 3192S), anti-phospho eIf2α (Cell Signal 9721S), anti-eIf2α (Cell Signal 9722S), and anti-ATF4 (Cell Signal 11815S).

Blots were developed using SuperSignal West Pico chemiluminescent substrate (Pierce) and signals were detected using the VersaDoc system (model 3000, Bio-Rad) or Fuji. Quantification of band intensities was performed using Quantity One 4.5.2 software (Biorad). For specific detection of PrP^Sc^, 20 μg of protein were digested with 25 μg ml^-1^ proteinase K in 20 μl final volume of digestion buffer (0.5% wt/vol sodium deoxycholate and 0.5% vol/vol Nonidet P-40 in PBS) for 30 min at 37°C [11]. Loading buffer was added and samples were boiled at 95°C for 5 min to inactivate PK. PNGase F treatment was performed using a commercially available kit, according to the manufacturer’s protocol (New England Biolabs). In brief, 10 μg of proteins was treated with 2 μl denaturation buffer in a 20 μl sample volume and incubated for 15 min at 95°C. A reaction mixture containing 2.6 μl G7, 2.6 μl NP-40 (10%) and 0.5 μl PNGase was added to the samples and incubated for 2h at 37°C. Samples were then boiled in presence of loading dye, and subjected to western blot analyses.

### Immunofluorescence staining and quantification

For immunofluorescence staining, organotypic slices were rinsed twice in PBS and fixed in 4% formalin overnight at 4°C. After washing, membrane inserts were incubated in blocking buffer (0.05% vol/vol Triton X-100 and 3% vol/vol goat serum in PBS) for 1 h and incubated with primary antibodies diluted in blocking buffer at 4°C for 3 days. The primary antibodies and concentrations used were mouse anti-Neuronal Nuclei (NeuN, 1 μg ml^-1^, Serotec), and directly conjugated mouse anti-NeuN-Alexa^488^ (0.5 μg ml^-1^, Millipore). The primary antibodies were detected using Alexa-conjugated secondary antibodies (3 μg ml^–1^, Molecular Probes). Membrane inserts were washed four times with PBS and the counterstaining agent 4,6-diamidino-2-phenylindole (DAPI) (1 μg ml^–1^) was added during the third washing step. Membranes were cut and mounted with fluorescent mounting medium (Dako) on a glass slide. Images were taken at identical exposure times with a fluorescence microscope (BX-61, Olympus) equipped with a cooled black/white CCD camera using a 4x objective. Morphometric analyses were performed to quantify the area of immunoreactivity using image analysis software analySIS vs5.0.

### Viability and ROS assays

For PI incorporation, slices were rinsed with PBS and incubated for 30 min with PI (5 μg ml^-1^). Live images were recorded at 5x magnification using a fluorescent microscope (Axiovert 200) equipped with a cooled CCD camera using a 5x objective and processed using image analysis software analySIS vs5.0.

The lucigenin conversion assay was carried out at room temperature (RT). Inserts containing 5–10 slices each were washed in PBS and lysed with a 30G syringe in Krebs-Ringer solution supplemented with complete mini protease inhibitor cocktail (Roche). 50 μl of tissue lysate was transferred to a 96-well white microplate containing 175 μl assay solution and 0.25 μl lucigenin (10 mM) per well. Background measurements were performed using a chemiluminescence reader prior to the addition of 50 μl NADPH (1 mM) to each well. Subsequently, the NADPH-dependent signal was read and subtracted. Data are presented as relative light unit mg^-1^ total protein (each bar: average of 4 inserts ± s.d.).

For DHE conversion measurements, slices were inoculated, incubated for 40 dpi, and washed twice in GBSS. They were then incubated in GBSS containing DHE (10 μg ml^-1^). After 20 minutes of incubation at RT, 3 images/slice were recorded by live fluorescence microscopy using Axiovert 200 equipped with a cooled CCD camera and using a 10x objective. Three images were recorded per slice in three individual folia of the cerebellum. Fluorescence of DHE oxidation products was assessed by morphometry using constant thresholds.

*In vivo* assessment of ROS production followed the protocol described by Murakami et al [36]. Thirty minutes prior to euthanasia, mice were injected intraperitoneally with 200 μl DHE, and brain tissue was homogenized in 50 mM KH_2_PO_4_, 1 mM EGTA, and 150 mM sucrose. Fluorescence of DHE oxidation products was measured in 250 μl of 2% (w/v) homogenates using a fluorimeter with Ex/Em 485/585nm and a cutoff of 570. Relative fluorescence units were normalized to protein concentration.

### Scrapie cell assay in endpoint format (SCEPA)

Prion-susceptible neuroblastoma cells (subclone N2aPK1) [22,23] were exposed to 300 μl cerebellar slice homogenates, with 6 replicates per dilution, in 96-well plates for 3 days. Cells were subsequently split three times 1:10 every 3 days. After the cells reached confluence, 25’000 cells from each well were filtered onto the membrane of ELISPOT plates, treated with PK (0.5 μg ml^–1^ for 90 min at 37°C), denatured, and infected (PrP^Sc^) cells were detected by immunocytochemistry using alkaline phosphatase-conjugated POM1, mouse anti-PrP, and an alkaline phosphatase-conjugated substrate kit (Bio-Rad). We performed serial ten-fold dilutions of experimental samples in cell culture medium containing healthy mouse brain homogenate. Scrapie-susceptible PK1 cells were then exposed to dilutions of experimental samples ranging from 10^–4^ to 10^–7^ (corresponding to homogenate with a protein concentration of 10 μg ml^-1^ to 0.01 μg ml^-1^), or to a 10-fold dilution of RML or healthy mouse brain homogenate. Samples were quantified in endpoint format by counting positive wells according to established methods [22,23].

### Statistical analysis of COCS

One-way ANOVA with Tukey’s post-hoc test for multi-column comparison, or Dunnett’s post-hoc test for comparison of all columns to a control column, were used for statistical analysis of experiments involving the comparison of three or more samples. Paired Student’s t-test was used for comparing two samples. Results are displayed as the average of replicates ± s.d.

### Microarray analyses

COCS were exposed to RML/NBH or POM1/IgG for various time points; for each time point and treatment, four cell culture inserts (n = 4) with 10 slices were used. RNA was extracted from 10 slices per insert using TRIZOL reagent (Invitrogen, USA) and purified with RNeasy columns (Qiagen, USA). Quality was assessed using BioAnalyzer (Agilent US). Labeled cDNA was fragmented and hybridized to GeneChip Mouse Genome 430 2.0 Array (Affymetrix, USA) which contains 45 000 probe sets. The data was analyzed with R/Bioconductor. Preprocessing and normalization was done using the RMA algorithm [37] and differential expression was assessed using the limma [26] package.

### Nanostring

The nCounter Analysis system has been introduced previously [38]. Briefly, for each gene of interest, two sequence-specific probes are designed. The probes are complementary to a 100-base region of the target mRNA. The first probe is covalently linked to an oligonucleotide containing biotin (capture probe), and the second probe is covalently linked to a color-coded molecular tag that provided the signal (reporter probe). Forty-nine probe pairs for test genes and control genes were contained in the nCounter CodeSet.

### Ethics statement

All mouse experiments were carried out according to Swiss law and conducted under the approval of the Animal Experimentation Committee of the Canton of Zurich (permits 200/2007, 90/2013 and 130/2008). The animal care and protocol guidelines were obtained from http://www.blv.admin.ch/themen/tierschutz/index.html?lang=en and strictly adhered by the experimenters and animal facility at the institution where the experiments were performed.

## Supporting Information

S1 Fig Characterization of GDL-exposed and prion-infected COCS over time. **(A-B)** COCS were exposed to POM1 for 8 h-10 days and assessed for ongoing cell death by PI incorporation (**A**) PI incorporation plateaued at 1–5 dpe, Untreated slices (ut), or slices exposed to pooled IgG, were used as controls (grey). (**B**) In prion-infected COCS (+), PI incorporation peaked at 38 dpi. Controls (-): exposure to non-infectious brain homogenate. Data were analyzed using a two-tailed t-test; *n* = 9 biological replicates.
(TIF)

S2 Fig ROS production and ER stress in wild-type COCS exposed to GDL. (**A**) wild-type COCS exposed for 7 or 14 days to either IgG or POM1 (286 nM each) were treated with DHE, and fluorescent particles per 10x view field were counted. At 7 and 14 dpe a significant ROS burst was detected (n = 30 replicates). (**B**) COCS were cultured for 14 days, treated with POM1 or IgG, and harvested at 7 dpe. Western blots were prepared from lysates and probed for p-PERK, PERK, p-eIF2α, eIF2α and ATF4. Densitometry (normalization to the non-phosphorylated form or actin for ATF4) revealed a trend towards increased p-PERK, p-eIF2α. (**C**) COCS treated as in (B) were harvested at 14 dpe. p-eIF2α was significantly upregulated, and ATF4 close to statistical significance (p = 0.07). Densitometry data were analyzed using a two-tailed t-test; *n* = 3 biological replicates.
(TIF)

S3 Fig Western blot analyses of prion-infected COCS treated with ascorbate. Lanes 1–11 of immunoblots A, B and C are reproduced from a previous study [13] for convenience. RML-infected *tg*a*20* COCS were exposed to Asc starting at 21 dpi, and harvested at 39 dpi. Homogenates of treated COCS were digested with proteinase K (PK) (**A**), left untreated (**B**), or treated with PNGase F (**C**). Western blots were probed with POM1 to detect PrP^Sc^, total PrP, and total unglycosylated PrP (full-length and C1/C2 proteolytic fragments). Ascorbate and E64d only marginally affected the C1/C2 processing of PrP **(C)**, and did not alter the PK digestion pattern of PrP^Sc^ (A). Sc: brain homogenate of RML-infected *tg*a*20* mouse. FL: full-length PrP. **(D)** Densitometry revealed no changes in FL or cleavage into the C1 fragment upon ascorbate treatment in RML-infected slices. Only cleavage into the C2 fragment was reduced upon Asc treatment. Densitometry data are presented as average ± s.d. and were analyzed by one-way ANOVA with Dunnett post-hoc test, *n* = 3 biological replicates.
(TIF)

S4 Fig Viability testing of COCS treated with various tool compounds. COCS prepared from *tg*a*20* mice were treated with pooled IgG at 67 nM for 10–14 days (upper graph) or with non-infectious brain homogenate (NBH) for 43 days (lower graph) in the presence of the compounds listed in the figure. Grey dots: no compounds were added. None of the compounds tested affected the viability of COCS, as assessed by NeuN morphometry; *n* = 9 biological replicates. The effects of compounds labeled with “†” on IgG-exposed COCS and zVAD labeled with “§” on NBH-infected COCS were reported previously [15], [13] and are reproduced here for convenience.
(TIF)

S5 Fig Scatter plot comparing genes of selected signaling pathways between POM1-exposed and RML-infected COCS. Scatter plots comparing the expression level of genes that are involved in specific signaling pathways found in RML-infected and POM1-exposed COCS. The 45 days RML time point (x-axis) and the different time points of POM1 exposure (y-axis) are represented. The correlation coefficient (cor) is indicated above each graph. These five signaling pathways have been described to be activated upon prion infection. **(A)** Autophagy, **(B)** CCL2 signaling, **(C)** ER stress response, **(D)** ERK inhibition and **(E)** TNF-ROS-Caspase 3 cascade.
(TIF)

S6 Fig Heat maps of the expression pattern of genes involved in the five pathways analyzed: Autophagy, CCL2 signaling, ER stress response, ERK inhibition and the TNF-ROS-Caspase 3 cascade. Downregulated (blue) and upregulated (red) genes (log2ratio) are shown for RML vs NBH (left; purple) and POM1 vs IgG (right; green). Four replicates are depicted for each condition. The specific pattern of upregulated and downregulated genes observed at 45 days post RML infection can also be found with increasing exposure time of POM1.
(TIF)

S7 Fig Comparison of the pathogenetic features of prion- and GDL-induced neurotoxicity. All investigated parameters are congruent in both models, except for the time line of toxicity (which is explained by the large differences in the concentration of the active principle).
(TIF)

S8 Fig PrP^Sc^ immunoblots of brain homogenates from AcHyT treated mice. Brain homogenates from *tg*a*20* mice infected with 22L and optionally treated with AcHyT were digested with proteinase K and probed with the antibody POM1 for PrPSc. Negative control: non-infectious brain homogenate (NBH).
(TIF)

S1 Table Lists of upregulated and downregulated genes at the different time points in RML-infected COCS. Differential expression was defined as fold change ≥ 2; *p* value < 0.01 vs NBH-exposed COCS.
(XLSX)

S2 Table Lists of upregulated and downregulated genes at different time points in POM1-exposed COCS. Differential expression was defined as fold change ≥ 2; *p* value < 0.01 vs IgG exposed COCS.
(XLSX)

S3 Table Comparison of the gene lists. Genes upregulated and downregulated at 45dpi (RML), 3 dpe and 7 dpe (POM1).
(XLSX)

S4 Table Top 40 activated pathways using GeneGo MetaCore analysis. Lists of the upregulated and downregulated genes at 45dpi for RML, and 3dpe and 7dpe for POM1 were uploaded. The top 40 pathways for POM1 at 3dpe and top 50 for POM1 at 7dpe were computed. Nine of the top 10 of upregulated pathways at 45dpi for RML were either activated at 3dpe or 7dpe POM1.
(XLS)

S5 Table Comparison of values of fold change measured in microarray and nanostring. (XLSX)

S6 Table Summary table of the tool compounds used and their biological targets. (DOCX)
