# Correction: Prion Infections and Anti-PrP Antibodies Trigger Converging Neurotoxic Pathways

**DOI:** 10.1371/journal.ppat.1004808

**Published:** 2015-04-14

**Authors:** Uli S. Herrmann, Tiziana Sonati, Jeppe Falsig, Regina R. Reimann, Paolo Dametto, Tracy O'Connor

The authors inadvertently omit Dr. Hubert Rehrauer in their Acknowledgements section.

The correct Acknowledgements section should be: We thank Dr. Edward Oakeley, Dr. Hubert Rehrauer, Rita Moos, Beata Sikorska, Dr. Cinzia Tiberi, Petra Schwarz, Ahmet Varol, Karina Arroyo, Clémence Tournaire and Mirzet Delic for technical help and Dr. Asvin Lakkaraju, Dr. Vijay Chandrasekar and Dr. Caihong Zhu for discussions.
