# Supplementary figures and images for: Prion Infections and Anti-PrP Antibodies Trigger Converging Neurotoxic Pathways
Source: PLoS Pathog. 2015 Feb 24;11(2):e1004662. doi: 10.1371/journal.ppat.1004662 (PMC4339193; doi:10.1371/journal.ppat.1004662)

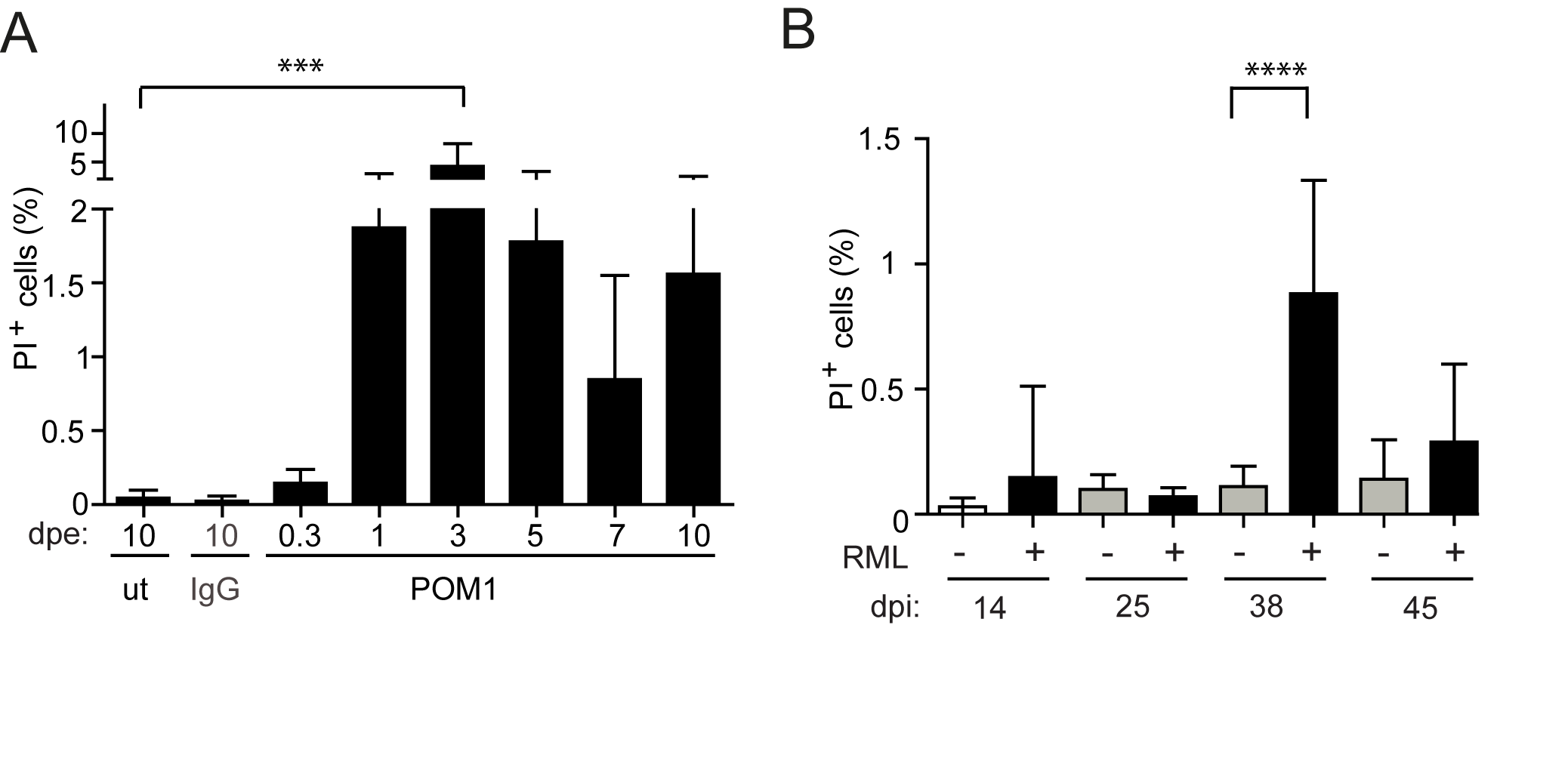

Supplement: S1 Fig — (A-B) COCS were exposed to POM1 for 8 h-10 days and assessed for ongoing cell death by PI incorporation (A) PI incorporation plateaued at 1–5 dpe, Untreated slices (ut), or slices exposed to pooled IgG, were used as controls (grey). (B) In prion-infected COCS (+), PI incorporation peaked at 38 dpi. Controls (-): exposure to non-infectious brain homogenate. Data were analyzed using a two-tailed t-test; n = 9 biological replicates. (TIF) [file ppat.1004662.s001.tif]

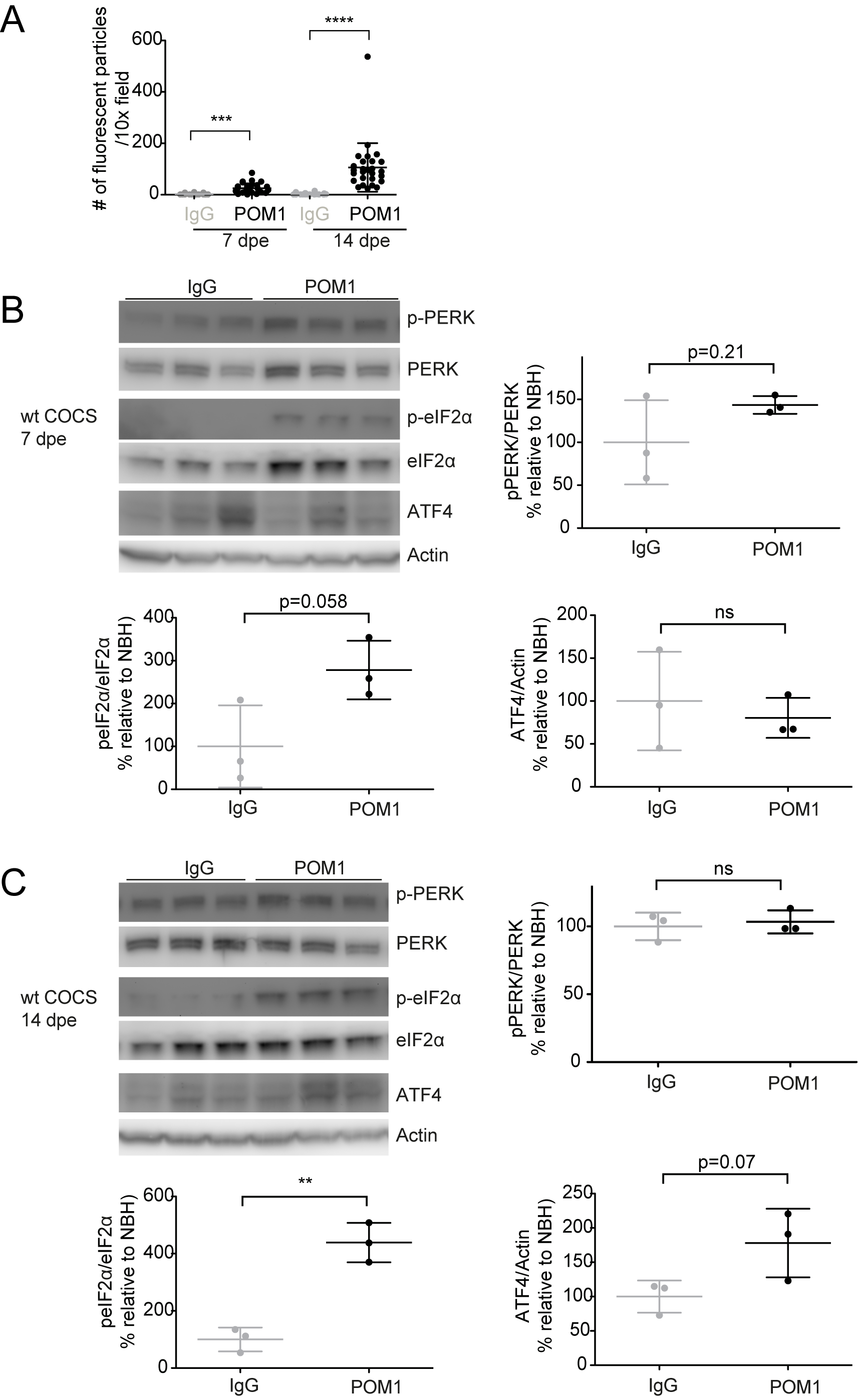

Supplement: S2 Fig — (A) wild-type COCS exposed for 7 or 14 days to either IgG or POM1 (286 nM each) were treated with DHE, and fluorescent particles per 10x view field were counted. At 7 and 14 dpe a significant ROS burst was detected (n = 30 replicates). (B) COCS were cultured for 14 days, treated with POM1 or IgG, and harvested at 7 dpe. Western blots were prepared from lysates and probed for p-PERK, PERK, p-eIF2α, eIF2α and ATF4. Densitometry (normalization to the non-phosphorylated form or actin for ATF4) revealed a trend towards increased p-PERK, p-eIF2α. (C) COCS treated as in (B) were harvested at 14 dpe. p-eIF2α was significantly upregulated, and ATF4 close to statistical significance (p = 0.07). Densitometry data were analyzed using a two-tailed t-test; n = 3 biological replicates. (TIF) [file ppat.1004662.s002.tif]

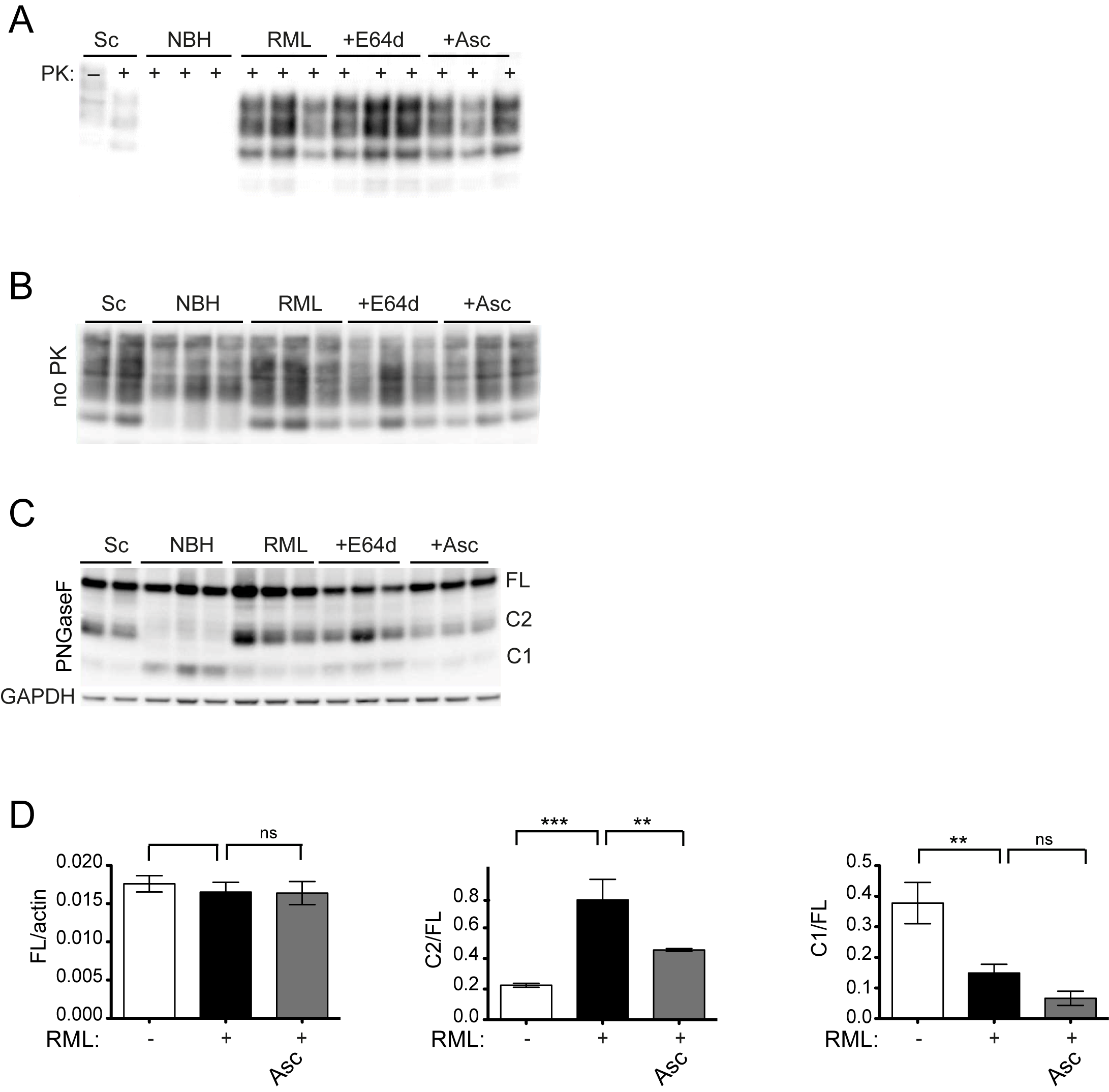

Supplement: S3 Fig — Lanes 1–11 of immunoblots A, B and C are reproduced from a previous study [13] for convenience. RML-infected tga20 COCS were exposed to Asc starting at 21 dpi, and harvested at 39 dpi. Homogenates of treated COCS were digested with proteinase K (PK) (A), left untreated (B), or treated with PNGase F (C). Western blots were probed with POM1 to detect PrPSc, total PrP, and total unglycosylated PrP (full-length and C1/C2 proteolytic fragments). Ascorbate and E64d only marginally affected the C1/C2 processing of PrP (C), and did not alter the PK digestion pattern of PrPSc (A). Sc: brain homogenate of RML-infected tga20 mouse. FL: full-length PrP. (D) Densitometry revealed no changes in FL or cleavage into the C1 fragment upon ascorbate treatment in RML-infected slices. Only cleavage into the C2 fragment was reduced upon Asc treatment. Densitometry data are presented as average ± s.d. and were analyzed by one-way ANOVA with Dunnett post-hoc test, n = 3 biological replicates. (TIF) [file ppat.1004662.s003.tif]

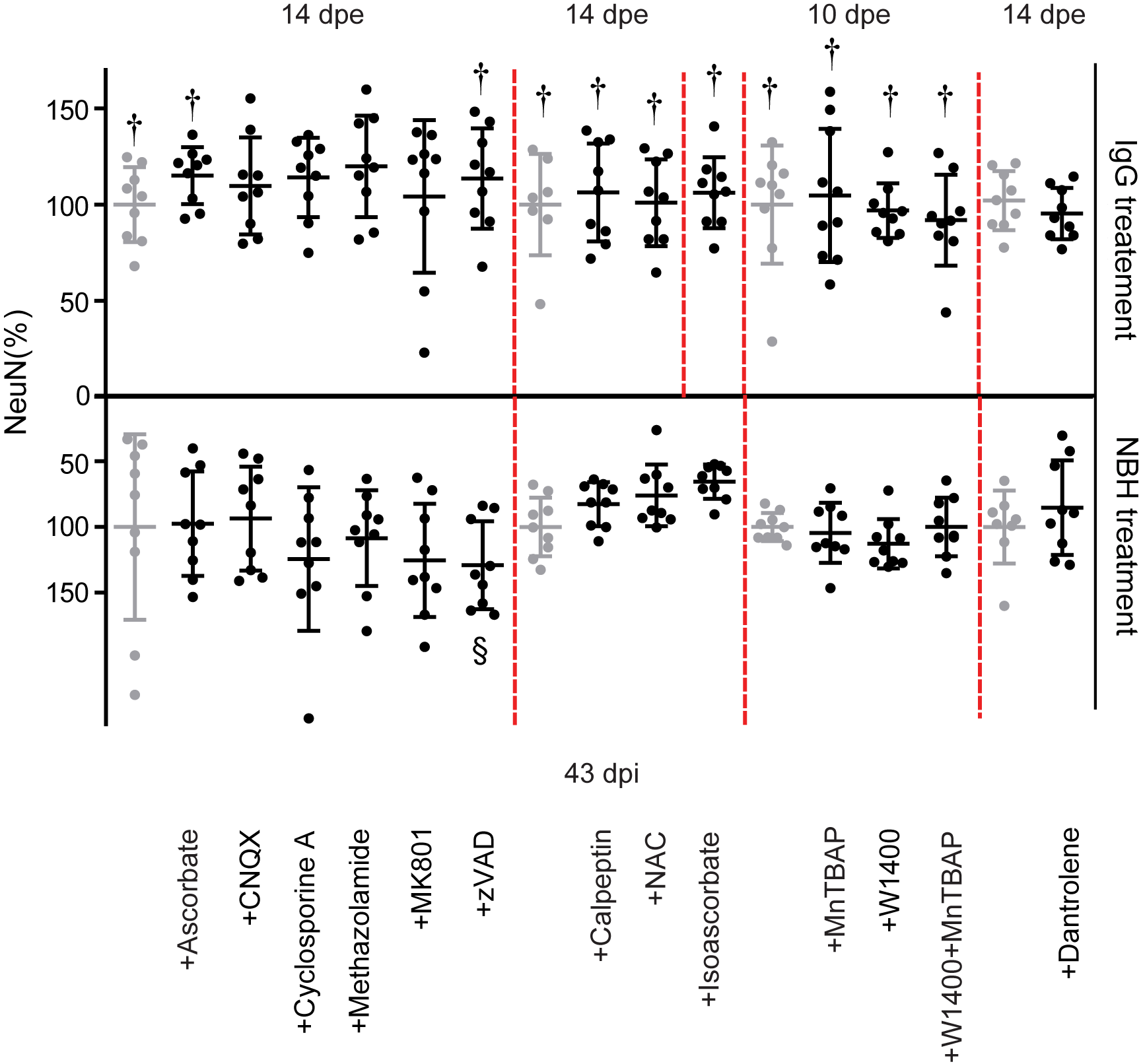

Supplement: S4 Fig — COCS prepared from tga20 mice were treated with pooled IgG at 67 nM for 10–14 days (upper graph) or with non-infectious brain homogenate (NBH) for 43 days (lower graph) in the presence of the compounds listed in the figure. Grey dots: no compounds were added. None of the compounds tested affected the viability of COCS, as assessed by NeuN morphometry; n = 9 biological replicates. The effects of compounds labeled with “†” on IgG-exposed COCS and zVAD labeled with “§” on NBH-infected COCS were reported previously [15], [13] and are reproduced here for convenience. (TIF) [file ppat.1004662.s004.tif]

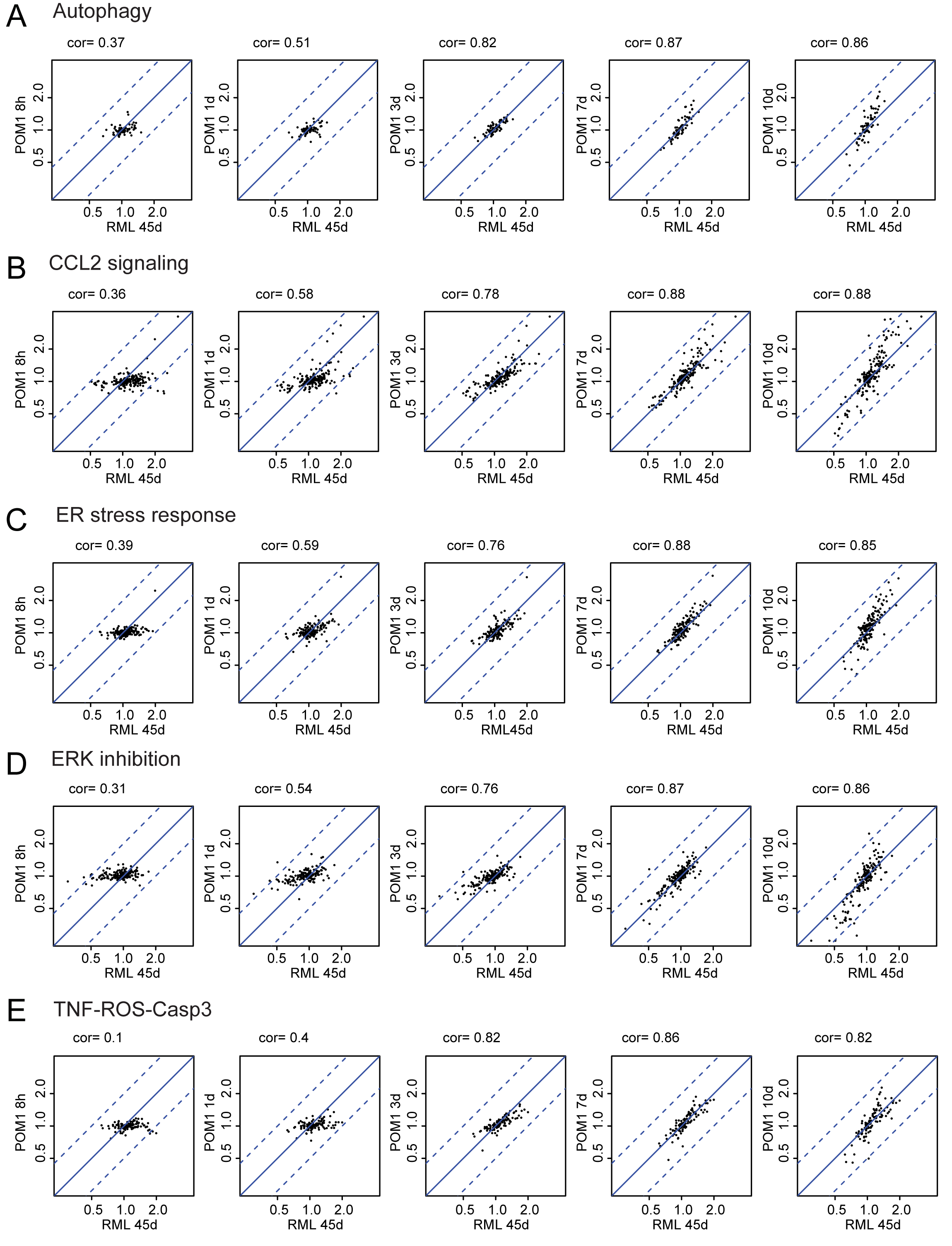

Supplement: S5 Fig — Scatter plots comparing the expression level of genes that are involved in specific signaling pathways found in RML-infected and POM1-exposed COCS. The 45 days RML time point (x-axis) and the different time points of POM1 exposure (y-axis) are represented. The correlation coefficient (cor) is indicated above each graph. These five signaling pathways have been described to be activated upon prion infection. (A) Autophagy, (B) CCL2 signaling, (C) ER stress response, (D) ERK inhibition and (E) TNF-ROS-Caspase 3 cascade. (TIF) [file ppat.1004662.s005.tif]

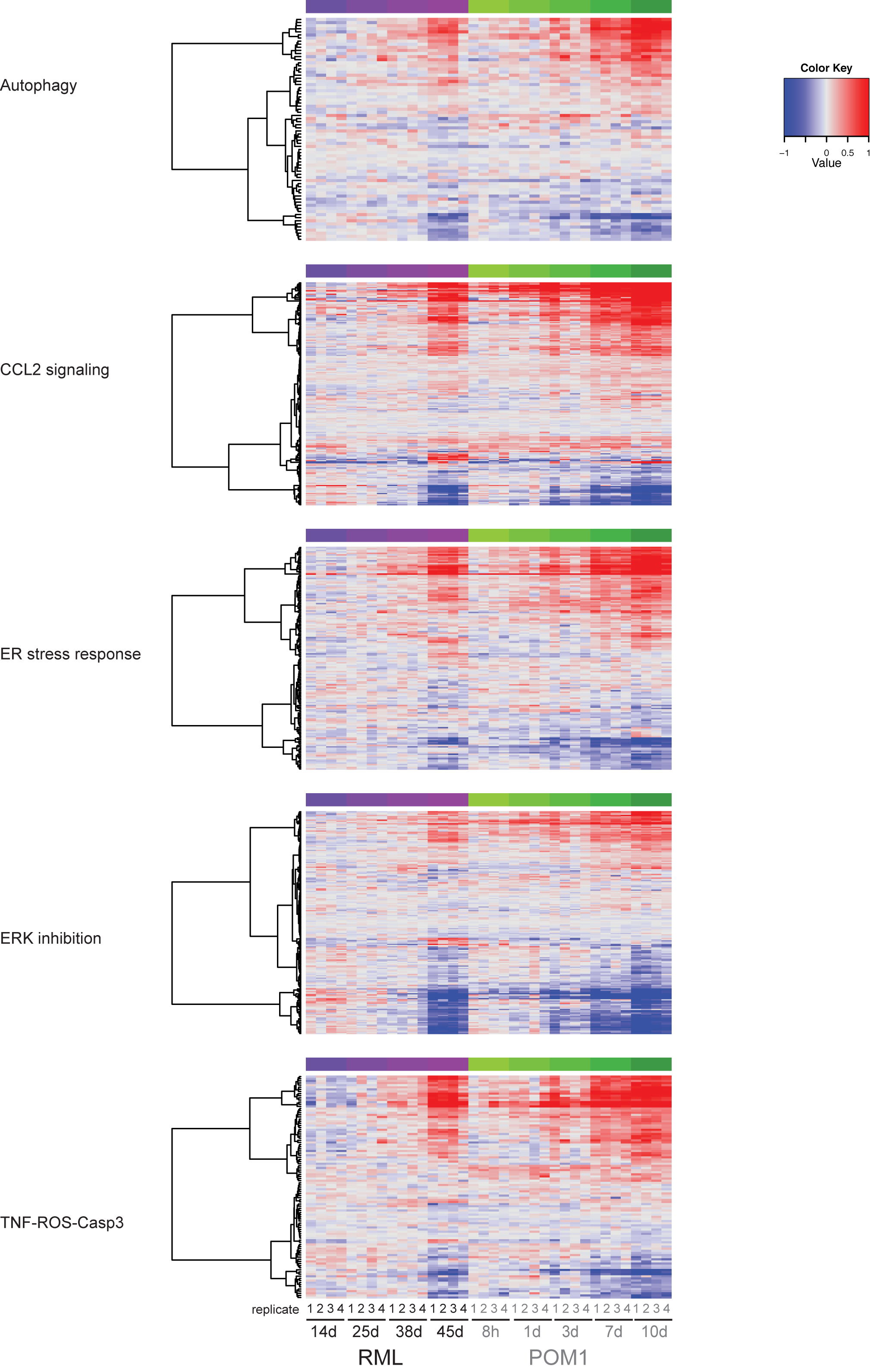

Supplement: S6 Fig — Downregulated (blue) and upregulated (red) genes (log2ratio) are shown for RML vs NBH (left; purple) and POM1 vs IgG (right; green). Four replicates are depicted for each condition. The specific pattern of upregulated and downregulated genes observed at 45 days post RML infection can also be found with increasing exposure time of POM1. (TIF) [file ppat.1004662.s006.tif]

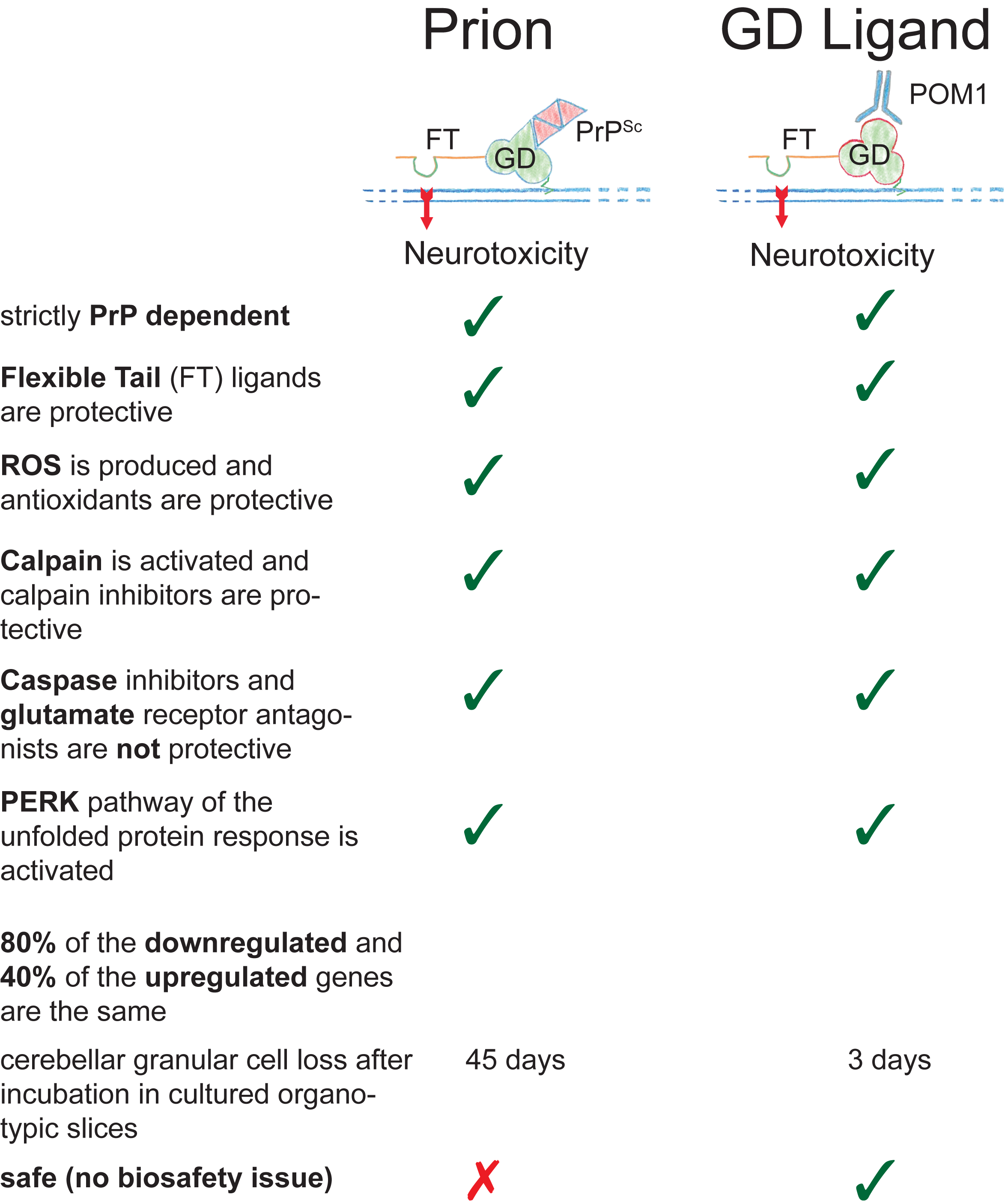

Supplement: S7 Fig — All investigated parameters are congruent in both models, except for the time line of toxicity (which is explained by the large differences in the concentration of the active principle). (TIF) [file ppat.1004662.s007.tif]

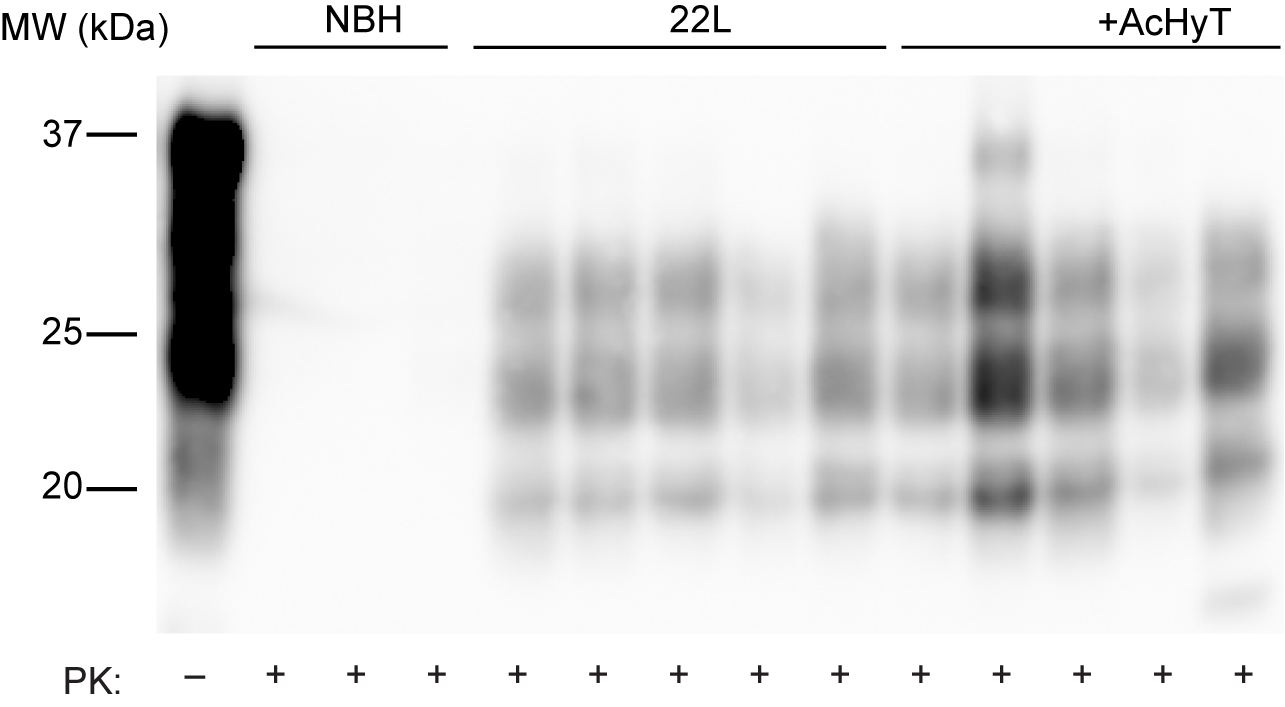

Supplement: S8 Fig — Brain homogenates from tga20 mice infected with 22L and optionally treated with AcHyT were digested with proteinase K and probed with the antibody POM1 for PrPSc. Negative control: non-infectious brain homogenate (NBH). (TIF) [file ppat.1004662.s008.tif]
