# Supplementary material for: Prion Infections and Anti-PrP Antibodies Trigger Converging Neurotoxic Pathways
Source: PLoS Pathog. 2015 Feb 24;11(2):e1004662. doi: 10.1371/journal.ppat.1004662 (PMC4339193; doi:10.1371/journal.ppat.1004662)
Supplement: S6 Table — (DOCX) [file ppat.1004662.s014.docx]

### Table S6. Summary table of the tool compounds used and their biological targets.

| Compound | Primary target  (according to published data) | Neuroprotection | |
| --- | --- | --- | --- |
|  |  | POM1 | prion |
| 1400W | Nitric oxide synthase 1 and 2 [[36](#_ENREF_36)] | no* | no |
| Ascorbate | ROS scavenging [[37](#_ENREF_37)] | yes* | yes |
| Calpeptin | Cysteine-proteases: Calpains and cathepsins [[38](#_ENREF_38),[39](#_ENREF_39)] | yes* | yes |
| CNQX | Ionotropic AMPA and kainate receptors [[13](#_ENREF_13),[40](#_ENREF_40)] | no | no |
| Cyclosporine A | Mitochondrial membrane permeability transition pore [[41](#_ENREF_41)] | no | no |
| Dantrolene  DPI | Ryanodine receptor [[42](#_ENREF_42)]  Inhibitor of ROS-producing electron transporters, such as the NADPH oxidase enzymes (NOX) [[43](#_ENREF_43)] | no  n/a* | no  n/a |
| Isoascorbate | ROS scavenging[[44](#_ENREF_44)] | yes* | yes |
| Methazolamide | Mitochondrial membrane permeability transition pore [[45](#_ENREF_45)] | no | no |
| MK801 | NMDA receptor [[13](#_ENREF_13),[40](#_ENREF_40),[43](#_ENREF_43),[46](#_ENREF_46)] | no | no |
| MnTBAP | Superoxide and H_2_O_2_ scavenging [[40](#_ENREF_40)] | yes* | yes |
| N-acetyl-cysteine | ROS scavenging [[47](#_ENREF_47)] | yes* | yes |
| zVAD-FMK | Broad-spectrum caspase inhibitor [[38](#_ENREF_38)] | no* | no^§^ |

n/a: not amenable to assessment due to toxic effects

^§,*^: data that have been published elsewhere [[9](#_ENREF_9),[11](#_ENREF_11)] and are reproduced here for convenience.
